# Probiotic *Lactobacillus acidophilus* provides an adjuvant effect to an orally administered chitosan nanoparticle-based *Salmonella* subunit vaccine in broilers against *Salmonella* Enteritidis infection

**DOI:** 10.3389/fmicb.2026.1898587

**Published:** 2026-10-15

**Authors:** Sara Dolatyabi, Shreeya Sharma, Comfort Olaitan Shekoni, Raksha Suresh, Sudhir Yadav, Jennifer Schrock, Ronna Wood, Gowtham Avverahalli Shivalingaiah, Alok Dhakal, Megan Tenney, Tonima Rahman, James Sanko, Ramon Zegpi Lagos, Khaled Abdelaziz, Gourapura J. Renukaradhya

**Affiliations:** 1 Center for Food Animal Health, Department of Animal Sciences, College of Food, Agricultural, and Environmental Sciences, The Ohio State University, Wooster, OH, United States; 2 Department of Animal and Veterinary Science, Clemson University, Clemson, SC, United States; 3 Clemson University School of Health Research (CUSHR), Clemson, SC, United States

**Keywords:** adjuvant, broiler chicken, chitosan nanoparticle, gut microbiome, *Lactobacillus*, mucosal immunity, *Salmonella* enteritidis, vaccine

## Abstract

*Salmonella enterica* serovar Enteritidis (SE) is a prominent zoonotic pathogen that contaminates poultry meat and eggs, leading to widespread foodborne illness in humans. Despite better biosecurity measures and available commercial vaccines, SE foodborne illness from broiler meat remains a challenge, necessitating a novel vaccination strategy. This study investigated the adjuvant efficacy of probiotic *Lactobacillus acidophilus* to a mannose-conjugated chitosan nanoparticle-based *Salmonella* subunit nanovaccine encapsulated with outer membrane proteins (OMP) and flagella antigens (FLA) of SE. Broiler chicks (*n* = 17–22 birds/group across six treatment groups) were administered the probiotic *in ovo* (embryonic day 18) and post-hatch (days 2, 4, and 6), and oral vaccination was performed at days 3 and 21 and challenged with SE at day 35. The physical nature of the nanovaccine includes zeta potential (34.62 ± 1.34 mV), particle size (286.5 ± 6.87 nm), and polydispersity index (0.45 ± 0.04), ensuring stability and suitability for mucosal delivery. Our results demonstrated that the probiotic-nanovaccine combination synergistically elicited specific immune responses associated with a 1.2 log_10_ reduction in cecal SE colonization at day post challenge 4 compared with a commercial live-attenuated vaccine. Immunological analysis revealed an elevated frequency of activated B-cells, increased specific secretory IgA in the intestines and bile. Cytokine profiling in the cecal tonsils showed upregulation of Th1/Th17-associated cytokines (IFN-γ/IL-17) gene expression and transition to regulatory cytokine IL-10, indicative of a balanced immune response. Furthermore, 16S rRNA sequencing of cecal contents showed that the probiotic-nanovaccine did not alter overall microbiota diversity but significantly shifted microbial community composition (*R*^2^ = 0.378, *p* = 0.001). Probiotic-supplemented groups were enriched with short-chain fatty acid producing *Clostridiales*, including *Blautia, Butyricicoccus*, and *Christensenellaceae R-7 group*, whereas the commercial vaccine group exhibited higher abundance of *Escherichia/Shigella* and reduced *Lactobacillus*. These findings underscore the potent adjuvant effect of *L. acidophilus* in enhancing the immunogenicity and protective efficacy of an oral *Salmonella* nanovaccine, while favorably modulating the gut microbiome. This integrated probiotic-nanovaccine approach represents a promising, antibiotic-free paradigm for mitigating salmonellosis in poultry, with implications for reducing zoonotic transmission, incidence of foodborne illness, and antimicrobial resistance.

## Introduction

*Salmonella enterica* serovar Enteritidis (SE) is one of the most prevalent non-typhoidal *Salmonella* serovars causing human salmonellosis, transmitted primarily through contaminated poultry meat and eggs (Scallan et al., 2011; Vencia et al., 2015). The Centers for Disease Control and Prevention (CDC) estimates 1.35 million infections, 26,500 hospitalizations, and 420 deaths annually in the United States alone (Majowicz et al., 2010), with severe outcomes implicated in children under five, the elderly, and immunocompromised individuals (European Food Safety Authority European Centre for Disease Prevention and Control, 2023). The economic burden is substantial, with costs in Australia alone exceeding $811 million yearly due to gastroenteritis-related productivity losses and healthcare expenses (Ford et al., 2019). In the European Union, SE remains the dominant serotype in zoonotic reports, linked to 8.9% of confirmed cases, predominantly from raw poultry (Tauxe, 1991).

SE establishes intestinal colonization in poultry, particularly in the cecum, with consequent fecal shedding and environmental persistence (Nazir et al., 2025). While biosecurity measures, such as sanitized housing, feed monitoring, and hygiene protocols, are critical for reducing SE prevalence, they are often cost-prohibitive and insufficient for complete eradication (Liu et al., 2016). Antimicrobial use for prophylaxis has been curtailed due to rising resistance concerns, exemplified by multidrug-resistant serovars like *S*. Heidelberg (MacLennan et al., 2014). This convergence of foodborne illness, agricultural antimicrobial use, and zoonotic transmission underscores the value of a One Health approach. Vaccination has therefore emerged as a cornerstone strategy for controlling salmonellosis in poultry flocks (Shaji et al., 2023).

Conventional *Salmonella* vaccines each carry their own tradeoff: live vaccines administered mimic natural infection and induce robust cell-mediated and mucosal immunity, but they pose risks of virulence reversion and environmental contamination, prompting FDA restrictions on pre-slaughter use (Chagas et al., 2024); inactivated vaccines are safer but require labor-intensive parenteral administration, yielding suboptimal secretory IgA (sIgA) responses critical for mucosal barrier protection (Apaka, 2024); and subunit vaccines using antigens like outer membrane proteins (OMP) and flagellin (FLA), offer enhanced safety and cross-serovar protection via conserved epitopes, but necessitate adjuvants and delivery vehicles to surmount gastrointestinal barriers (Guo et al., 2023).

Nanotechnology-based delivery systems, such as mannose-conjugated chitosan nanoparticles (NPs) address these challenges by protecting antigens from enzymatic degradation, promoting mucoadhesion, and targeting mannose receptors on antigen-presenting cells (APCs) in gut-associated lymphoid tissues (GALT) (Renu and Renukaradhya, 2020). Chitosan's cationic nature facilitates electrostatic interactions with negatively charged mucosal surfaces, enhancing antigen uptake by M-cells and dendritic cells (Diaz Carrasco et al., 2019). Prior studies from our laboratory have validated mannose-chitosan NP-based *Salmonella* subunit vaccines in eliciting innate and adaptive immune responses in broilers and layers (Renu and Renukaradhya, 2020). Adjuvants further amplify vaccine efficacy, with *Mycobacterium smegmatis* whole cell lysate (WCL), a non-pathogenic alternative to *M. tuberculosis* components, providing immunostimulatory elements like N-glycolyl muramyl dipeptide (MDP) and trehalose-6,6-dimycolate (TDM) that have been shown to activate innate pathways without inducing granulomatous reactions (Hansen et al., 2014).

Probiotics, defined by the World Health Organization as live microorganisms conferring health benefits when administered adequately, represent an additional adjuvant strategy (Lone et al., 2022). *Lactobacillus* species, in particular, modulate gut microbiota, competitively exclude microbial pathogens, enhance epithelial barrier integrity, produce antimicrobial metabolites (e.g., bacteriocins, organic acids), and stimulate mucosal immunity through cytokine and antimicrobial peptide modulation in the intestine and GALT (El-Shall et al., 2019). *Lactobacillus acidophilus* was selected for this study based on published evidence that it modulates gut mucosal immunity and competitively excludes enteric pathogens, properties that make it well suited as a probiotic adjuvant against *Salmonella* colonization in poultry. Seeding the developing gastrointestinal tract of chicks with probiotic bacteria via *in ovo* and early post-hatch administration has been shown to reduce susceptibility to pathogens (Rychlik, 2020). Furthermore, in adult birds, while gut microbial dysbiosis exacerbates *Salmonella* colonization, the increased abundance of beneficial taxa like short-chain fatty acid (SCFA) producers (e.g., *Butyricicoccus* and *Blautia*) has been shown to mitigate this effect by enhancing gut barrier function and anti-inflammatory responses (Lépine et al., 2018). The combined potential of probiotic and nanoparticle-adjuvanted vaccine strategies to synergistically improve mucosal immunity against SE in poultry remains unexplored. To our knowledge, this is the first study to evaluate a dual-adjuvant strategy combining a live probiotic with a mannose-chitosan nanoparticle-based subunit vaccine against *Salmonella* in poultry and performed the 16S rRNA sequencing to characterize concurrent microbiome shifts. The objective of this study was to determine whether the probiotic *L. acidophilus* potentiates the mucosal immunogenicity of mannose-chitosan nanoparticle-based oral *Salmonella* subunit vaccine, thereby reducing SE colonization and promoting favorable microbiome shifts in broiler chickens.

## Materials and methods

### Ethics statement and animal welfare

All experimental procedures involving animals were conducted in strict accordance with ethical guidelines and were approved by The Ohio State University Institutional Animal Care and Use Committee (IACUC 2016A00000060-R2). The study adhered to the principles outlined in the Animal Welfare Act, the Guide for the Care and Use of Laboratory Animals, and the American Veterinary Medical Association (AVMA) Guidelines for the Euthanasia of Animals. Broiler chickens were housed in biosafety level 2 (BSL-2) facilities at the Center for Food Animal Health (CFAH), The Ohio State University, Wooster, OH, USA (40.80 °N, 81.94 °W; elevation ~301 m above sea level). The site experiences a humid continental climate, characterized by warm, moderately wet summers and cold snowy winters. Birds were offered *ad libitum* commercial feed and fresh water under BSL-2 husbandry conditions throughout the study. Birds were monitored daily for signs of distress, and euthanasia was performed humanely using carbon dioxide asphyxiation.

### Bacterial strains and culture conditions

A field-isolated strain of *Salmonella enterica* serovar Enteritidis (SE; phage type 13a), resistant to nalidixic acid (20 μg/mL) and novobiocin (20 μg/mL), was used for nanovaccine antigen preparation, and challenge experiments. The strain was cultured in Luria-Bertani (LB) broth (Difco, BD Biosciences) at 37 °C with shaking at 150 rpm until reaching mid-logarithmic phase (optical density at 600 nm [OD_600_] ≈ 0.6–0.8). Bacterial concentrations were determined by serial dilution plating on Xylose Lysine Tergitol-4 (XLT4) agar plates supplemented with the aforementioned antibiotics.

The probiotic strain, *Lactobacillus acidophilus* (*L. acidophilus*-P42), was cultured in de Man, Rogosa, and Sharpe (MRS) broth (Difco, BD Biosciences) under anaerobic conditions at 37 °C for 18–24 h. Viable counts were determined on MRS agar plates incubated anaerobically. For administration, bacterial pellets were resuspended in sterile phosphate-buffered saline (PBS; pH 7.4) to achieve a concentration of 10^6^ colony-forming units (CFU)/dose for *in ovo* injection and 10^7^ CFU for post-hatch supplementation.

### Antigen extraction and purification

The OMP and FLA were extracted from SE cultures as previously described, with minor modifications (Suresh et al., 2025). For OMP extraction, SE was cultured in Tryptic Soy Broth (TSB) until the culture reached an optical density (OD_600_) of approximately 1.0. Bacterial cells were harvested by centrifugation and subsequently washed with Tris-HCl buffer. The pellet was stored at −80 °C overnight. Following thawing, bacteria were heat-inactivated by incubation at 75 °C for 10 min. The heat-killed bacterial suspension was sonicated and centrifuged at 8,000 x g for 10 min. The resulting supernatant was ultracentrifuged at 108,726 x g for 30 min. The pellet was washed in Tris-HCl buffer containing 2% Triton X-100, incubated for 30 min at room temperature, and further ultracentrifuged at 100,000 x g for 2 h. The final pellet containing purified OMP was resuspended in PBS. For FLA extraction, SE was cultured in Brain Heart Infusion (BHI) broth at 37 °C under static conditions. The bacterial cells were harvested by centrifugation, washed once with PBS, and treated with 3M potassium thiocyanate for 2 h at 4 °C with continuous magnetic stirring. The suspension was ultracentrifuged at 35,000 x g for 30 min. The supernatant containing FLA protein was collected and dialyzed initially against PBS for 12 h, followed by an additional 12 h dialysis in Milli-Q water. Protein integrity and expected molecular weight profiles were confirmed by sodium dodecyl sulfate-polyacrylamide gel electrophoresis (SDS-PAGE) with Coomassie Brilliant Blue staining as described previously (Suresh et al., 2025), revealed characteristic bands for OMP (e.g., 35-55 kDa porins) and FLA (≈55 kDa FLA monomer). Protein concentrations were determined using the Pierce BCA Protein Assay Kit (Thermo Fisher Scientific), with bovine serum albumin as the standard.

### SDS-PAGE analysis

Protein purity was assessed by SDS-PAGE as described previously (Renu et al., 2018). Briefly, samples were mixed with an equal volume of 2X Laemmli sample buffer containing DTT (Sigma, St Louis MO) as the reducing agent and denatured at 95 °C for 5 min. Samples (10 μL) were resolved on two separate polyacrylamide gels of different acrylamide concentrations (12% and 5%) to have an optimum separation across the molecular-weight ranges of OMP and flagellin, run at 100 V for 1.5–2 h, and stained with Coomassie Brilliant Blue R-250.

### Nanovaccine formulation and physicochemical characterization

Chitosan used in our study was 75–85% deacetylated and mol. wt 50,000–190,000 Da based on viscosity. The mannose chitosan NPs formulation encapsulating SE OMP and FLA was prepared using the ionic gelation method as described previously (Zhao et al., 2012; Renu et al., 2021; Han et al., 2020). FLA was incorporated into two distinct compartments of the nanoparticle to serve complementary immunological roles. An encapsulated fraction combined with equal amount of OMP prior to ionic gelation was protected within the nanoparticle core to preserve antigen integrity during transit. A separate surface-displayed fraction of FLA was added after TPP crosslinking which was surface adsorbed on to the NPs. This surface attachment which happens by electrostatic interaction (positive charge of chitosan with negative charge of FLA) promotes engagement of FLA with TLR5 on mucosal antigen-presenting cells, enhancing the particle uptake by prolonged retention of particles on the mucosal surfaces facilitating interaction of particles with Peyer's patches and microfold (M) cells in the intestines (Salman et al., 2009; Renu et al., 2020). Briefly, 50 mg of lyophilized mannose-conjugated chitosan was dissolved in 50 mL of Milli-Q water under continuous magnetic stirring, and the pH was adjusted to 4.3. To this solution, 5 mg of pooled OMP and FLA (2.5 mg each) dissolved in 5 mL of 10 mM MOPS buffer (pH 7.4) was added so that both antigens were entrapped within the core during gelation. Subsequently, 12.5 mg of TPP dissolved in 25 mL of Milli-Q water was added dropwise to crosslink the nanoparticles (chitosan:TPP mass ratio of 4:1). Finally, 1.25 mg of FLA prepared in 1 mL of 10 mM MOPS buffer was added dropwise to coat the surface of preformed nanoparticle. The resulting NPs were collected as a pellet via centrifugation at 10,976 x g for 30 min.

The entrapment efficiency of proteins, both inside and on the surface of the NP formulation, was evaluated by measuring the unentrapped protein present in the supernatant using the Bicinchoninic Acid (BCA) assay, and was calculated as follows: Entrapment efficiency (%) = [(total protein added – unentrapped protein in supernatant) / total protein added] × 100. Each 500 μL oral vaccine dose contained 10 μg OMP and 10 μg FLA (20 μg total antigen) encapsulated within the mannose-chitosan nanoparticle core, plus a surface coating of FLA (1.25 μg). Ten μg WCL-NP adjuvant was used. Earlier we demonstrated that approximately 50% of added flagellin binds to the surface of OMP + FLA entrapped nanovaccine (Renu et al., 2020) (data not shown). A similar protocol was used for WCL-NP adjuvant formulation as described previously.

### Experimental design and bird management

Broiler eggs were obtained from a commercial hatchery and incubated under standard conditions (37.5 °C, 60% humidity, automatic turning every 3 h) until hatch. The study was divided into two phases to optimize probiotic administration and evaluate vaccine efficacy.

#### Phase 1: optimization of probiotic delivery

Eggs were randomly allocated to five treatment groups using simple randomization without blocking, with 12 eggs assigned to each group (Table 1). Prior to *in ovo* inoculation, all eggs were candled to confirm viability of embryo. On embryonic day 18 (ED18), *in ovo* administrations were performed using a sterile 23-gauge needle inserted into the amniotic fluid (identified by candling) to deliver 100 μL of probiotic (10^6^ CFU/dose as whole culture or bacterial cells suspended in PBS) or PBS (mock). Hatchability was monitored, and hatched birds were transferred to floor pens in isolators. Post-hatch administrations occurred on days 2, 4, and 6 after hatch via orally (1 x 10^7^ CFU/100 μL). Birds were reared until day 10 post-hatch, when subsets (*n* = 9–11/group) were euthanized for tissue collection (cecal tonsils, bursa of Fabricius and spleen). The timeline is depicted in Figure 1. Sample sizes were determined based on prior studies using similar challenge models. Personnel performing sample collection, tissue processing and results were blinded to treatment group allocation.

**Table 1:** Bird groups in phase-1 trial.

Groups	Immunization	Total birds	Probiotic supplementation	Necropsy
1	Mock	9	N/A	Day 10
2	Pre-hatch (bacterial cells)	11	ED18	Day 10
3	Post-hatch (bacterial cells)	10	day-post- hatch 2, 4, 6	Day 10
4	Pre and post-hatch (bacterial cells)	11	ED18, day-post- hatch 2, 4, 6	Day 10
5	Pre and post-hatch (suspension)	11	ED18, day-post- hatch 2, 4, 6	Day 10

**Figure 1:**
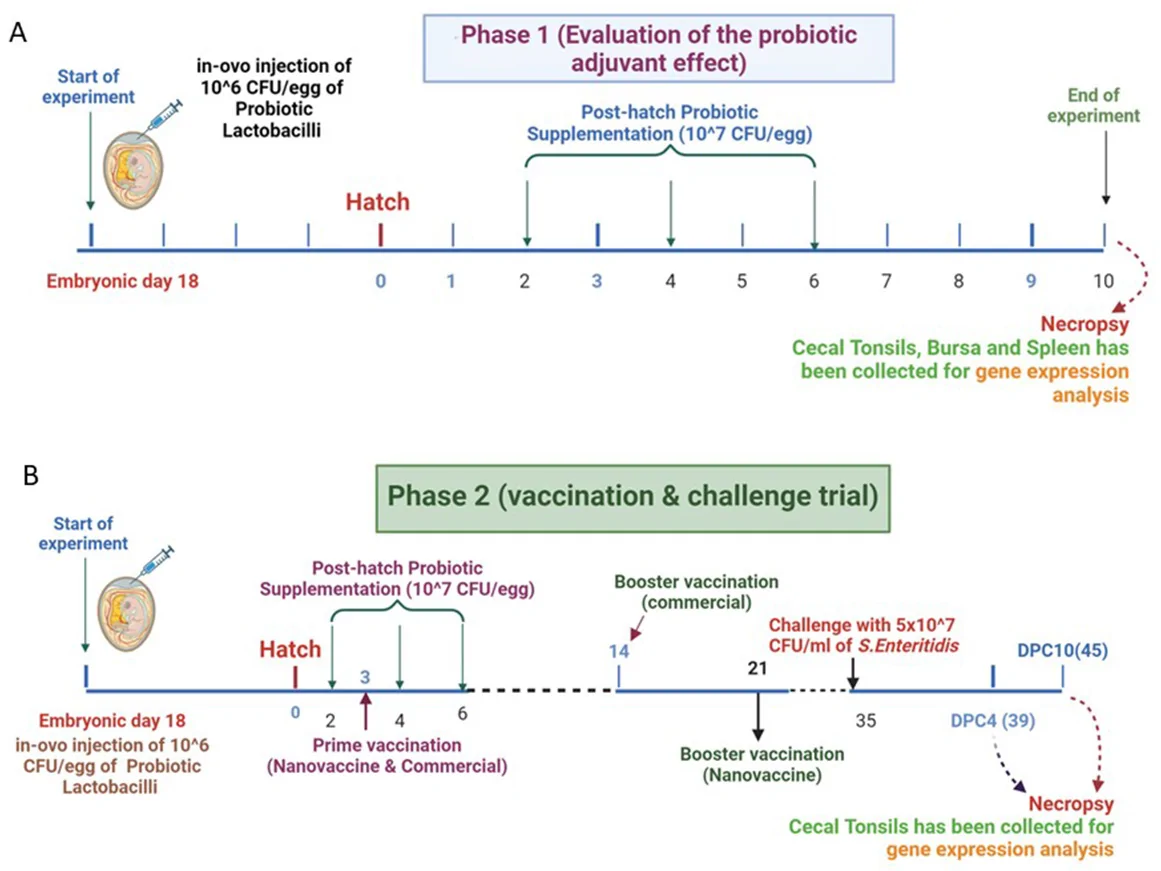
**(A)** Phase 1; Evaluation of the probiotic adjuvant effect project timeline. Bird groups: 1. Mock; 2. Pre-hatch (pellet); 3. Post hatch (pellet); 4. Pre and post hatch (pellet); 5. Pre and post hatch (suspension) **(B)**. Phase 2; Vaccination and challenge trial timeline.

#### Phase 2: vaccination and challenge trial

Based on Phase 1 results, Group 4 birds elicited superior immune priming with pre- and post-hatch and was therefore selected as the probiotic administration regimen carried out in Phase 2 trial. Hatched chicks were allocated to six treatment groups: Group 1, mock (no probiotic, no vaccine, and no challenge); Group 2, mock + challenge; Group 3, WCL-Sal-nanovaccine + challenge; Group 4, probiotic only + challenge; Group 5, WCL-Sal-nanovaccine + probiotic + challenge; and Group 6, commercial vaccine + challenge (Table 2). *L. acidophilus* was administered *in ovo* (1 × 10^6^ CFU/100 μL) at ED18 and days 2, 4, and 6 post-hatch orally (1 × 10^7^ CFU/100 μL). Oral nanoparticle vaccination (500 μL/dose) was given at days 3 and 21 post-hatch via orally. Each vaccine dose contained 10 μg each of OMP and FLA encapsulated and a surface coating of flagella in mannose chitosan-NPs, along with 10 μg of the adjuvant WCL-NP per dose. The commercial vaccine (Poulvac^®^ ST; Zoetis) was administered according to the manufacturer's instructions. Birds were orally challenged at age 35 days with 5 × 10^7^ CFU/bird of the same field-isolated SE strain (phage type 13a) described above, in 1 mL saline. Subsets (*n* = 8–13/group) were euthanized at 4 and 10 days post challenge (DPC), and samples collected included: blood (serum); cloacal swabs, small intestinal washes, and bile for antibody analysis; spleen for flow cytometry of lymphocyte populations; and cecal tonsils for cytokine gene expression analysis, with cecal contents used for bacterial enumeration. The sample collection timeline is shown in Figure 1.

**Table 2:** Bird groups in phase-2 trial.

Groups	Immunization	Total birds	DPC4	DPC10
1	Mock	20	10	10
2	Mock + challenge	21	10	11
3	WCL-Sal-Nanovaccine + challenge	17	9	8
4	Probiotics only +challenge	20	8	12
5	WCL-Sal-Nanovaccine + Probiotic + challenge	22	13	9
6	Commercial vaccine + challenge	20	10	10

## Immunological response assays

### Enzyme-linked immunosorbent assay (ELISA) for antigen-specific antibodies

OMP- and FLA-specific IgY and IgA antibodies were quantified in serum (1:500 dilution), cloacal swab (1:2), small intestinal wash (1:4), and bile (1:800) samples. High-binding 96-well plates (Corning) were coated in duplicate with pre-titrated OMP and FLA at 50 ng/well for IgY determination and OMP at 50 ng and FLA at 400 ng/well for sIgA determination. The antigen was diluted in coating buffer (50 mM Carbonate-Bicarbonate, pH 9.6) and added to each well at 50 μL/well, incubated overnight at 4 °C. Plates were washed thrice with PBS-Tween 20 (0.05%; PBST), blocked with 5% non-fat dry milk in PBST for 1 h at 37 °C, and washed again. Sera, intestinal washes, or bile samples (50 μL/well) were added, and plates were incubated for 2 h at 37 °C.

Secondary antibodies, Goat Anti-Chicken IgA peroxidase labeled (Biorad, Kidlington, UK) or Goat Anti-Chicken IgY (H+L) peroxidase labeled (Southern Biotech, Birmingham, AL) at pre-titrated dilution, were diluted in 2.5% dry milk powder in PBS-T and added and incubated at room temperature for 2 h. Following three washes with PBS-T, 50 μL/well of a 1:1 mixture of peroxidase substrate solution B and 3,3′,5,5′-Tetramethylbenzidine (Thermo Fisher Scientific) was added. The plates were kept in the dark, and the reaction was halted by adding 50 μL of 1M phosphoric acid per well for 10–20 min depending on the color development. Optical density was measured at 450 nm (OD450) using a SpectraMax M5 microplate reader (Molecular Devices). Samples were assayed at single pre-optimized dilution rather than serial dilutions, and antibody levels are reported as background-subtracted OD450 values as a semi-quantitative relative measure rather than endpoint titers or absolute concentrations. Each value represents the mean OD450 of duplicate wells, with background subtracted using uncoated blocked wells treated with the chromogen.

### Quantitative real-time polymerase chain reaction (qRT-PCR) for cytokine gene expression

The cecal tonsils were collected in RNAlaterTM solution (Invitrogen by Thermo Fisher Scientific, Vilnius, Lithuania) and stored at −80 °C until processing. A small part of the cecal tonsils was cut and homogenized by adding TRIzol™ Reagent (Invitrogen by Thermo Fisher Scientific). Chloroform (200 μL) was added, mixed, and incubated for 8 min. Subsequently, the mixture was centrifuged to achieve phase separation. Following centrifugation, the upper aqueous phase containing RNA was collected, precipitated with isopropanol, washed twice with ethanol, and resuspended in RNase-free water. Purity of the RNA was assessed using a NanoDrop spectrophotometer (Thermo Scientific, Waltham, MA, USA) by measuring the absorbance ratios at 260/280 and 260/230 nm. RNA (2,000 ng) was used as the reverse transcription template for cDNA synthesis in a 20 μL reaction containing the reaction buffer. The RNA was denatured at 70 °C for 12 min using a thermocycler. Each reaction of c-DNA conversion contains 4 μL of M-MLV RT 5x buffer, 1 μL of 10mM dNTPs, 0.5 μg of Oligo dT 15 primer, 100 units of M-MLV Reverse transcriptase, 8 units of recombinant RNAasin ribonuclease inhibitor (Promega, Madison, WI, USA), and 2 μL of 0.1M DTT (Invitrogen by Thermofisher Scientific, Carlsbad, CA). The mRNA expression was quantified by the SYBR Green method using QuantStudio 5 (Applied Biosystems by Thermofisher Scientific). The reaction mixture comprised 10 μL of PerfeCTa SYBR Green SuperMix (Quantabio, Beverly, MA, USA), 1 μL of cDNA template (50 ng), and 2 μL of each forward and reverse primer, made up to a final volume of 20 μL with RNase-free water. Primers for chicken cytokines (IFN-γ, IL-17A, IL-4, and IL-10 TGF-β IL-10) and the housekeeping gene β-actin were used at optimized annealing temperatures (Table 3). Reaction conditions: 95 °C for 5 min (1 cycle), followed by 40 cycles of 94 °C/20 s and the respective annealing temperature for 32 seconds. Melting curve analysis confirmed specificity. Relative gene expression was calculated using the 2^(−ΔΔCt)^ method, normalized to β-actin and mock controls. Reference-gene stability was confirmed by the low variability of raw β-actin Ct values across all cecal tonsil samples (mean 13.6 ± 1.1 Ct; coefficient of variation 8.3%) and by the absence of a significant difference among groups and between sampling time points, supporting its suitability as a normalizer.

**Table 3:** Sequences and annealing temperatures of different primers used for RT-qPCR.

Primer	Annealing temperature	Primers sequences
β-actin	58°C	F: CAACACAGTGCTGTCTGGTGGTA R: ATCGTACTCCTGCTTGCTGATCC
IL-4	56°C	F: AGC ACT GCC ACA AGA ACC TG R: CCT GCT GCC GTG GGA CAT
IL-10	55°C	F: CTG TCA CCG CTT CTT CAC CTR: ACT CCC CCA TGG CTT TGT A
IL-17A	55°C	F: TATCAGCAAACGCTCACTGG R: AGTTCACGCACCTGGAATG
IFN-γ	58°C	F: ACACTGACAAGTCAAAGCCGCACA R: AGTCGTTCATCGGGAGCTTGGC
TGFβ	60°C	F: CGGCCGACGATGAGTGGCTC R: CGGGGCCCATCTCACAGGGA

### Flow cytometry for lymphocyte phenotyping

The assay was performed as described previously (Han et al., 2020). Briefly, chicken splenocytes were isolated on the day of necropsy. Five million cells in 1 ml of complete RPMI medium (RPMI containing 2 mM L-glutamine, 10% fetal bovine serum, HEPES buffer and antibiotic-antimycotic) were seeded per well in a 48-well flat-bottom plate. The cells were stimulated with OMP and Flagella at 5 μg/mL each for 48 h to permit antigen-specific recall activation of memory lymphocytes; this concentration and duration were selected based on our earlier study (Han et al., 2020) to elicit measurable, antigen-specific upregulation of activation markers while avoiding non-specific stimulation or activation-induced cell death. The cells (1 x 10^6^) were transferred to a non-sterile 96-well round-bottom FACS plate, washed once with 200 μL FACS buffer/well and blocked for Fc receptors by using blocking buffer (1% normal mouse serum in FACS buffer) for 30 min at 4 °C. This step was followed by two washes with 1X PBS and live/dead staining using the Fixable Dead Cell Stain Kit (Invitrogen, ThermoFisher Scientific) at a 1:1000 dilution in PBS, with 50 μL/well for 30 min at 4 °C. The cells were then washed once in FACS buffer by centrifugation at 930 x g for 2 min and then subjected to surface staining using the specified anti-chicken lymphocyte-specific antibodies or their corresponding isotype control antibodies at previously optimized concentrations in a final volume of 50 μL for 30 min at 4 °C (Tables S1 and S2). Lymphocyte subsets were identified using the following surface markers: B-cells (CD3^−^Bu1^+^), T-helper cells (CD3^+^CD4^+^), and cytotoxic T-cells (CD3^+^CD4^−^CD8α^+^CD8β^+^). Compensation was performed using single-stained cell controls. Samples were acquired on a BD FACS Aria II machine, with a minimum of 50,000 live single-cell events collected per sample, and data were analyzed using FlowJo software v11.2 (BD Biosciences). Lymphocyte subset frequencies are expressed as the percentage of live lymphocytes. Cells were sequentially gated on the main lymphocyte cell population (FSC-A/SSC-A), single cells (FSC-A/FSC-H), and live cells (Fixable Dead Cell Stain), followed by identification of each lymphocyte subset. Positive gates for each subset were set using the corresponding isotype control antibodies-stained cells.

### Bacterial load enumeration

To quantify the SE load, ceca from each chicken were aseptically collected in Whirl-Pak bags during necropsy. The cecal contents were individually weighed and diluted to a 1:5 (w/v) ratio in PBS and manually squeezed. Serial tenfold dilutions were prepared and plated on Xylose Lysine Tergitol 4 (XLT4) agar plates supplemented with Nalidixic acid and Novobiocin, each at a concentration of 20 μg/mL. The plates were incubated at 37 °C for 24–36 h, and black colonies were enumerated. The *Salmonella* counts were expressed as CFU per gram of cecal content and log-transformed (log_10_ CFU/g) for statistical analysis.

### Microbiome analysis

#### Sample collection, DNA extraction, and library preparation

Cecal contents from DPC10 birds (*n* = 6/group) were snap-frozen and stored at −80 °C. Genomic DNA was extracted using the DNeasy PowerSoil Kit (Qiagen) following the manufacturer's protocol, including a 10 min bead-beating step to lyse microbial cells. DNA quality and quantity were assessed using a NanoDrop 2000 spectrophotometer (Thermo Fisher Scientific), ensuring 260/280 and 260/230 ratios >1.8 and concentrations >20 ng/μL. Samples were shipped on dry ice to the Clemson University Genomics and Bioinformatics Facility (CUGBF) for sequencing.

The procedures of Kozich et al. (2013) were followed to amplify the V3-V4 region of the 16S rRNA gene with slight modifications. Samples were amplified in 20 μL PCR reactions containing 1 μL DNA, 5 μL dual-index primers, 2 μL 10 × AccuPrime PCR Buffer II (Invitrogen), 0.15 μL AccuPrime HiFi polymerase, and 11.85 μL nuclease-free water. Thermal cycling conditions included an initial denaturation at 95 °C for 2 min, followed by 30 cycles of 95 °C for 20 s, 55 °C for 15 s, and 72 °C for 5 min, with a final extension at 72 °C for 10 min. Amplification success was confirmed via 2% agarose gel electrophoresis. Libraries were normalized (SequalPrep Normalization Plate Kit, Thermo Fisher), pooled, and quantified (NEBNext Library Quant Kit for Illumina). Paired-end sequencing was performed on an Illumina NextSeq 2000 using a P2 600-cycle reagent kit, generating an average of 455,812 reads per sample. Demultiplexing was conducted using Illumina DRAGEN (bcl2fastq v4.2.7). The sequences are stored in NCBI PRJNA1469281.

### Sequencing and bioinformatic processing

#### Bioinformatic analysis

Microbial community profiling was performed using 16S rRNA gene amplicon sequencing on the Illumina platform using paired-end sequencing chemistry. Raw paired-end FASTQ files were processed in QIIME2 v2022.8 using the DADA2 denoising pipeline. Demultiplexed paired-end sequences were imported into QIIME2, and sequence quality profiles were inspected using the demultiplexing summary visualization to determine truncation parameters prior to denoising. Forward and reverse reads were truncated at 285 bp and 240 bp, respectively, with no 5′ trimming applied (–p-trim-left-f 0, –p-trim-left-r 0) and QIIME2′s default plugin of 2.0. Amplicon sequence variants (ASVs) were inferred using the DADA2 plugin, which performs quality filtering, denoising, paired-end merging, and chimera removal.

Following denoising, a total of 4,302 ASVs were identified across 36 samples, generating 7,271,176 total reads. Samples had sequencing depths ranging from 124,655 to 582,245 reads per sample, with a mean sequencing depth of 201,977 reads/sample. Taxonomic classification was assigned using a pretrained SILVA v138 Naïve Bayes classifier trained on the 16S rRNA gene reference database (Quast et al., 2013). Sequences classified as chloroplasts, mitochondria, Archaea, Eukaryota, or unassigned taxa were removed prior to downstream analyses.

Alpha rarefaction analysis was performed to evaluate sequencing depth sufficiency, and a rarefaction depth of 124,655 reads per sample was selected based on the minimum sequencing depth retained across samples. Core diversity analyses were conducted in QIIME2.

### Statistical analysis

Data from immunological and bacterial load assays were analyzed using one-way analysis of variance (ANOVA) followed by Tukey's multiple comparisons *post-hoc* test in GraphPad Prism (v9.0). Within each cytokine assay, pairwise comparisons among groups were therefore corrected for multiple comparisons; a global correction across all cytokines, tissues, and time points was not applied. Fold changes in cytokine expression and antibody levels are presented as mean ± standard error of the mean (SEM).

Every bird was sampled individually for all immunological, bacteriological, and microbiome endpoints, and the individual bird served as the unit of measurement in the statistical analyses.

Downstream statistical analyses and visualization of microbiome data were analyzed in R software (v.4.4.0) using phyloseq (v.1.48.0), microeco (v.1.7.1), ggplot2 (v. 3.5.1), dplyr (v.1.1.4) and vegan (v.2.6) packages. ASVs with a total abundance ≤ 10 across all samples were removed, resulting in 1,242 retained ASVs for downstream analyses. Rarefaction curves were generated using the vegan package to confirm adequate sequencing coverage.

Microbial community composition and relative abundance analyses were performed using the microeco package. Taxonomic bar plots were generated at the genus, family, and phylum levels using the most abundant taxa across treatment groups. Alpha diversity indices were calculated in phyloseq, visualized as violin plots, and compared among groups using Kruskal Wallis test. Bray-Curtis distance matrices were used for beta diversity analyses and visualized using PCoA with 95% confidence ellipses. Permutational multivariate analysis of variance (PERMANOVA) was performed using the adonis2 function to assess microbial community differences among treatment groups. Homogeneity of group dispersions was evaluated using PERMDISP analysis. Differentially abundant taxa among treatment groups were identified using linear discriminant analysis effect size (LEfSe) implemented in the microeco package at the genus level with a linear discriminant analysis (LDA) threshold of 2.0 and Microbiome Multivariable Associations with Linear Models (MaAsLin2) with control group as the reference using the MaAsLin2 package in R. Features were filtered using a minimum abundance threshold of 0.0001 and minimum prevalence of 10%. Multiple testing correction was performed using the Benjamini-Hochberg method, and genera with q <0.25 were considered significantly differentially abundant.

## Results

### Nanovaccine characterization

The formulated WCL mannose conjugated chitosan-NP and Sal-nanovaccine had a zeta potential ranging from +30 to +35 mV, particle size ranged from 287 to 304 nm, a polydispersity index of 0.43 to 0.45, and an entrapment efficacy of cargo 71−76% (Table 4). The vaccine characterization was performed in triplicates in the Zetasizer. Enough of the nanovaccine required for this entire vaccine trial was prepared at once, aliquoted, combined with 5% sucrose as a cryoprotectant, freeze-dried, and stored at −20 °C. The Zetasizer was used at room temperature (25 °C), scattering angle around 173°, dispersant used was water, and sample concentration of 0.1 mg/ml.

**Table 4:** The physical nature of WCL mChitosan-NP and *Salmonella* nanovaccine.

Compound	Zeta potential (+mV)	*z*-average (nm)	Polydispersity index	Encapsulation efficiency %
WCL mChitosan-NP	30.47 ± 1.85	303.8 ± 8.54	0.43 ± 0.02	76.3
Sal-Nanovaccine	34.62 ± 1.34	286.5 ± 6.87	0.45 ± 0.04	71.1

### Phase 1: cytokine expression in lymphoid tissues following probiotic administration

*In ovo* inoculation of the probiotic *L. acidophilus* did not affect hatchability compared to the control group. In the cecal tonsils, IL-4 expression was significantly higher in group 2 (*in ovo* probiotic administration only) than that in the mock control group. The mRNA levels of IL-10, IL-17, IFN-γ, and TGF-β were numerically increased (not significant) across all the combinations of probiotic treatment compared to a mock control (Figure 2A).

**Figure 2:**
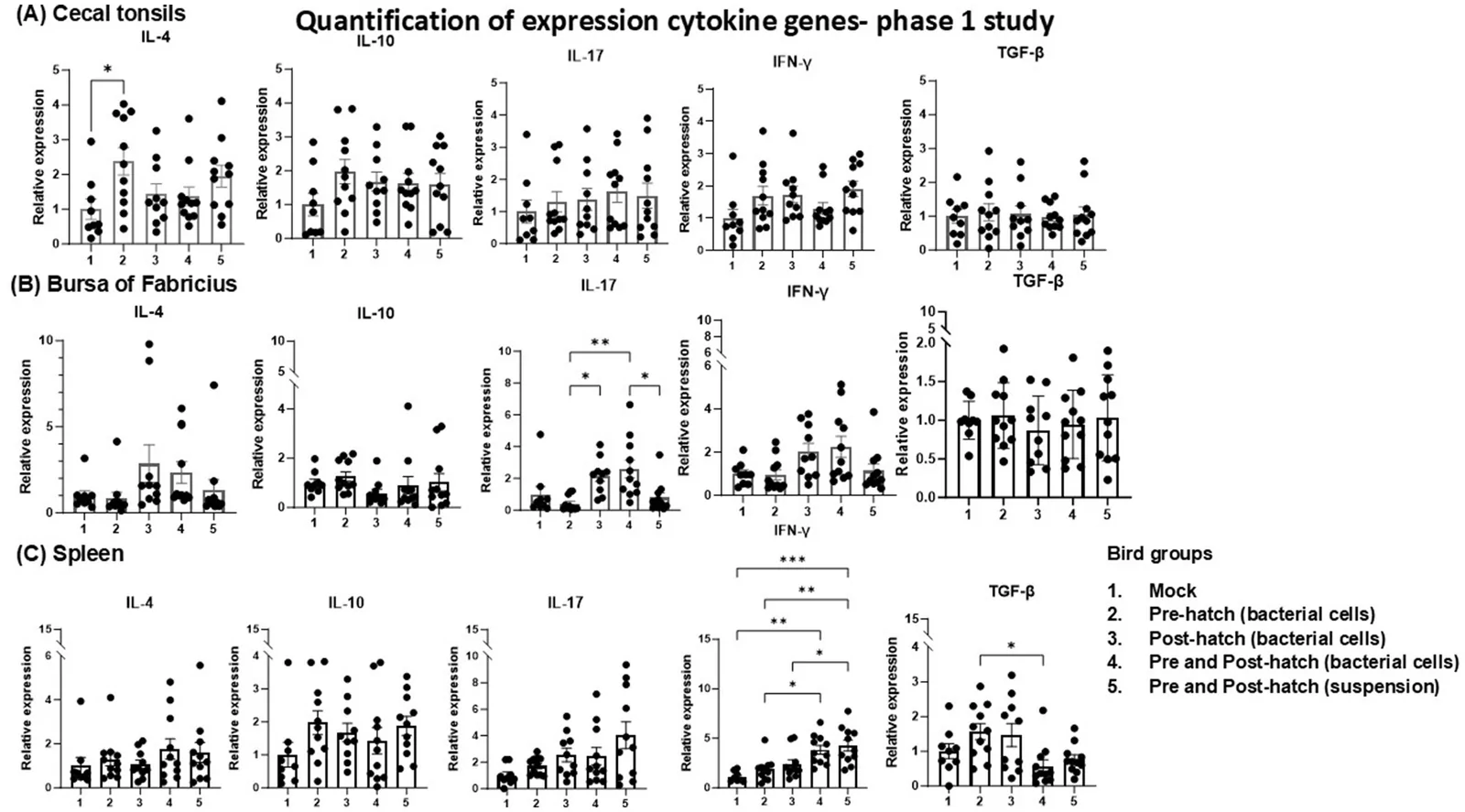
Cytokine gene expression: **(A)** Cecal tonsils of birds; **(B)** Bursa of Fabricius; **(C)** Spleen. Each bar represents the mean ± SEM of fold change in gene expression from 9–11 birds per treatment group. The *P-*values between groups were determined by one-way ANOVA followed by Tukey's multiple comparisons *post-hoc* test. Only significant comparisons are indicated in the figures.

In the bursa of Fabricius, the mRNA expression of IL-17 was significantly elevated and the levels of IL-4 and IFN-γ were increased in groups 3 and 4 compared to groups 2 and 5 birds. Expression levels of IL-10 and TGF-β did not have any differences among all the groups of birds (Figure 2B). In the spleen, IL-4 expression was slightly higher in groups receiving probiotics both pre- and post-hatch (groups 4 and 5) compared to other groups, whereas the IL-10 expression was numerically high in all treatment groups (Figure 2C). IL-17 expression was increased only in group 5 birds. IFN-γ expression was significantly elevated in groups 4 and 5 compared to other groups. A significant increase in TGF-β expression was observed in group 2 compared to group 4 (Figure 2C). Rather than uniform immune activation, in ovo and early post-hatch *L. acidophilus* inoculation was associated with selective, tissue-specific immune modulation, with significant increases in IL-4 (cecal tonsils), IL-17 (bursa of Fabricius), and IFN-γ (spleen), while other cytokines showed consistent but non-significant directional increase (Figure 2).

### Phase 2: antigen-specific sIgA and IgY responses following vaccination

To evaluate vaccine antigen-specific immune responses post-prime vaccination, the specific IgY responses against OMP and FLA in serum, and the sIgA responses against OMP and FLA in cloacal swabs were determined (Figure 3). Our data suggested that after prime vaccination (1 dose), there was no significant difference in both specific IgY and sIgA levels in all the vaccinated groups (Figure 3A).

**Figure 3:**
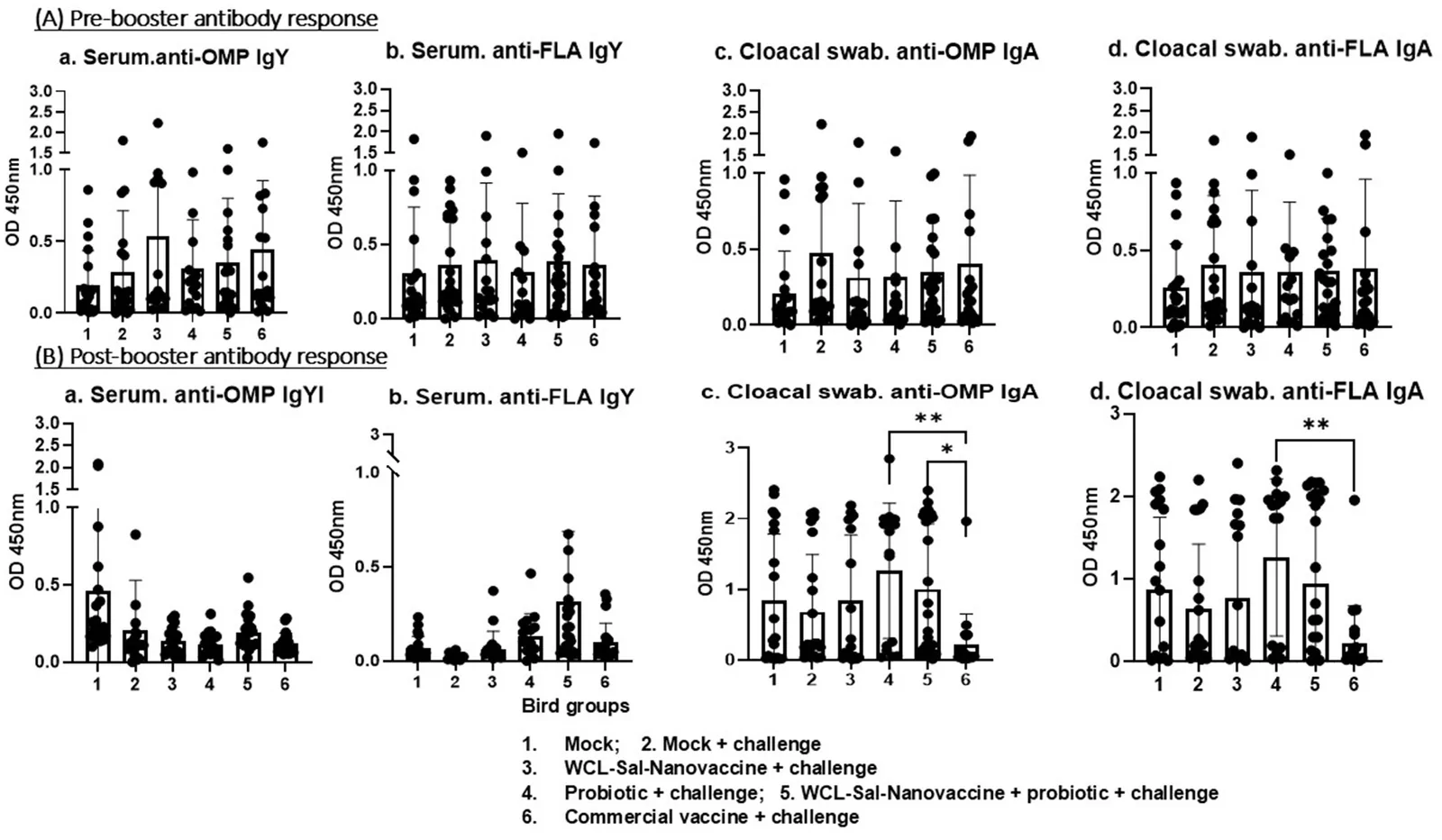
OMP and FLA specific antibody responses in serum and cloacal swab samples. **(A)** Pre-booster; **(B)** Post-booster. Each bar represents the mean ± SEM of 17–22 birds per group. The *P-*values between groups were determined by one-way ANOVA followed by Tukey's multiple comparisons *post-hoc* test. Only significant comparisons are indicated in the figures.

In the prime-boost (2 doses) vaccinated birds, there was also no significant difference in both specific IgY and sIgA levels in all the vaccinated groups, except for a numerical increase in the trends of antigen-specific sIgA levels in the cloacal swab of groups 4 and 5 compared to other groups was detected (Figure 3B).

At DPC 4, cloacal swab sIgA levels specific to OMP and FLA were significantly elevated in group 3 compared to group 6 (Figure 4Aa, Ab), whereas the specific sIgA and IgY levels in the small intestinal wash, bile and serum were not modulated in all the vaccinated groups (Figure 4A). At DPC10, in cloacal swab and bile, anti-FLA specific sIgA levels in group 3 were significantly elevated compared to group 6 (Figure 4B). The anti-OMP sIgA antibody levels in the small intestinal wash were significantly higher in group 5 compared to group 6 (Figure 4Bc).

**Figure 4:**
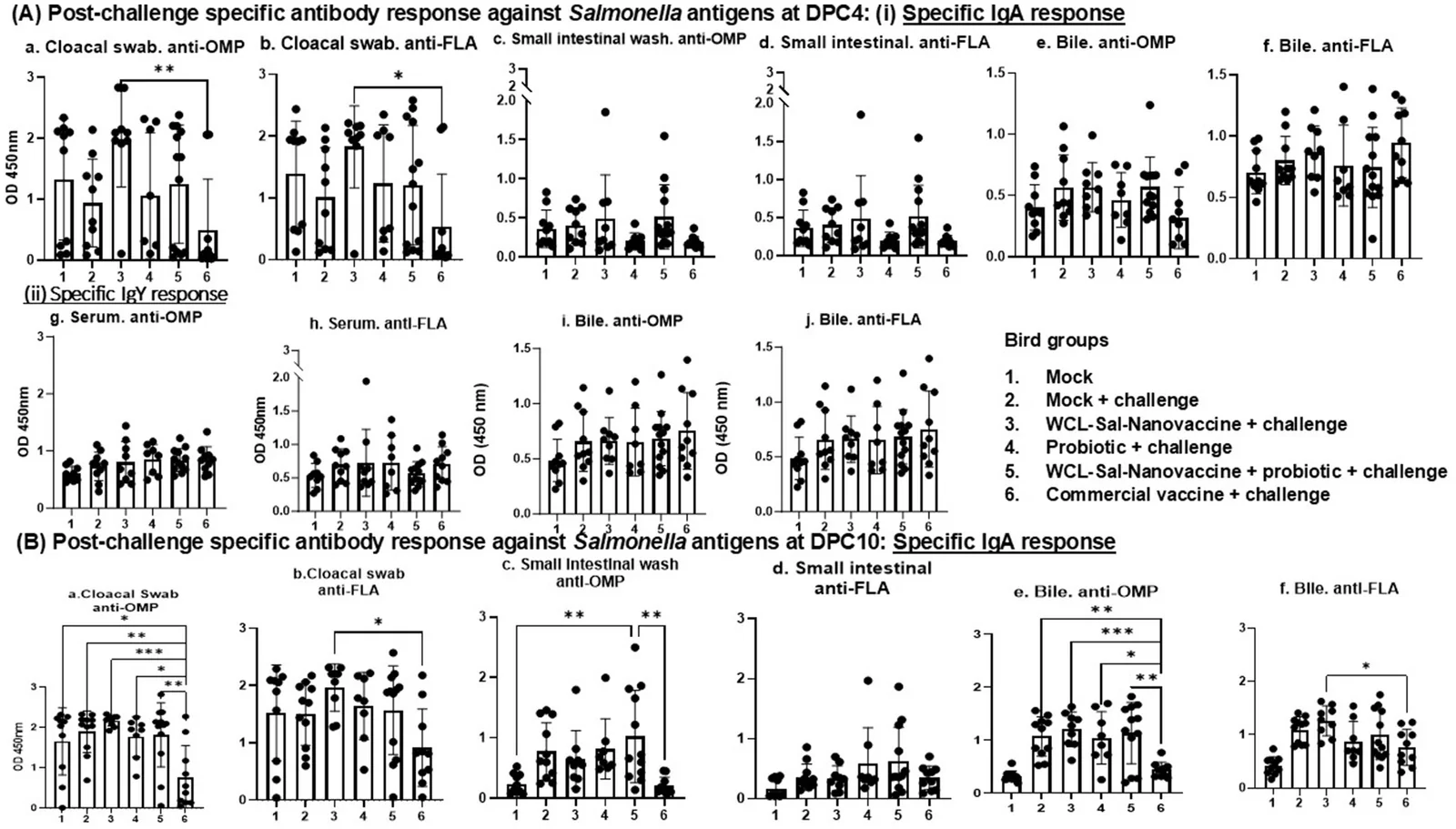
OMP and FLA specific antibody responses in: **(A)** Cloacal swab, small intestinal wash and bile samples at DPC4; and **(B)** Cloacal swab, small intestinal wash and bile samples at DPC10. Each bar represents the mean ± SEM of 8–12 birds per group. The *P-*values between groups were determined by one-way ANOVA followed by Tukey's multiple comparisons *post-hoc* test. Only significant comparisons are indicated in the figures.

#### Cecal cytokine gene expression after challenge

Gene expression data from cecal tonsils revealed notable changes in the cytokine expression profiles at DPC4 (Figure 5A). In particular, IL-4 mRNA expression showed a numerical increase in group 5 compared to all other groups. IL-10 gene expression levels were significantly higher in group 5 compared to groups 2, 3, and 6. Analysis of IL-17 gene expression showed significantly elevated levels in groups 2 and 5 compared to group 1. IFN-γ gene expression showed a numerical increase in group 5 compared to all other groups. No apparent differences in TGF-β expression were observed among all the bird groups at DPC4 (Figure 5A). At DPC 10, the gene expression analysis showed a numerical increase in IL-4 and IFN-γ levels in groups 4, 5, and 6, and IL-10 in group 4, compared to other groups. No apparent differences were observed among groups in IL-17 and TGF-β cytokine gene expression levels (Figure 5B).

**Figure 5:**
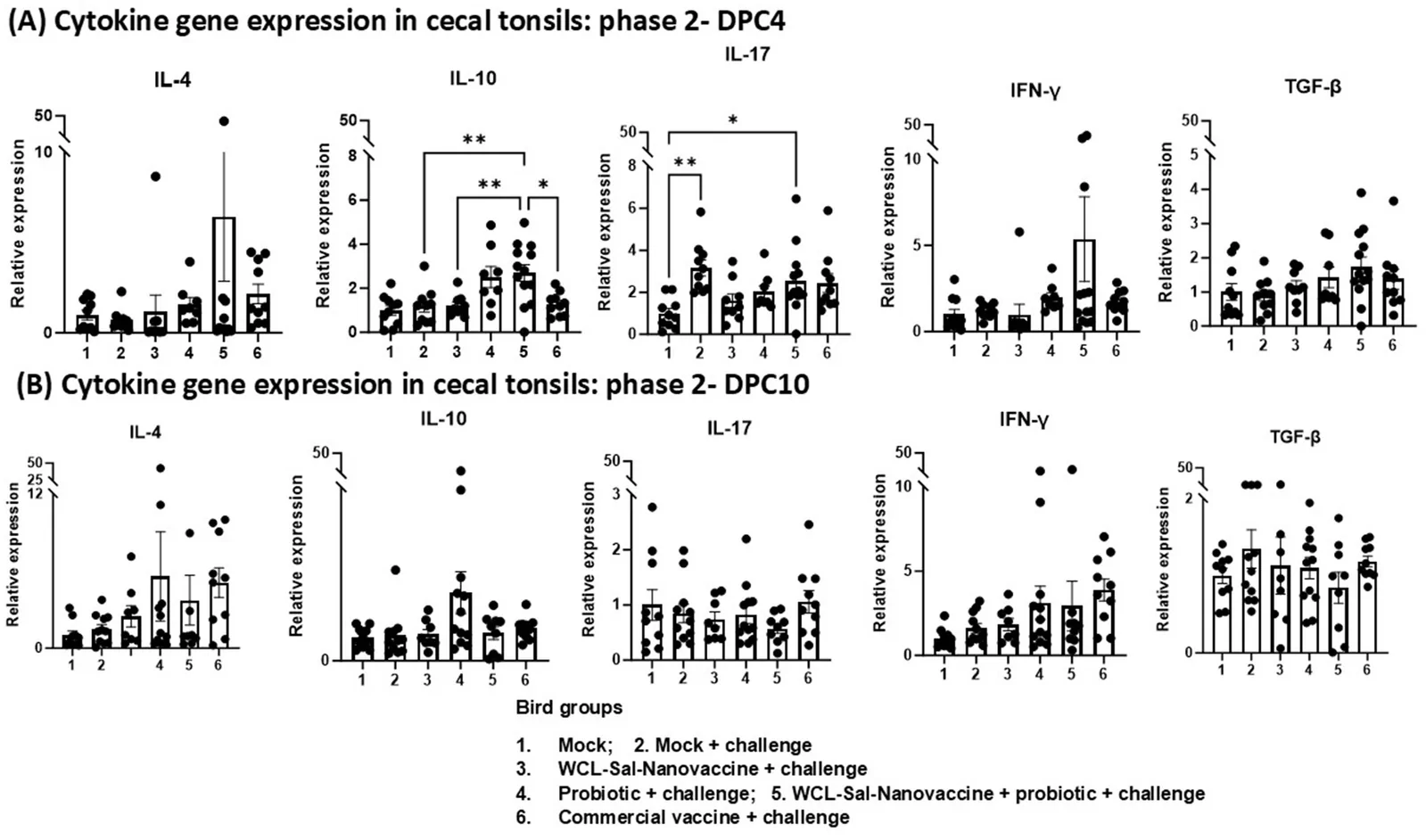
Cytokine gene expression in the cecal tonsils of birds at: **(A)** DPC4 and **(B)** DPC10. Each bar represents the mean ± SEM of 8–13 birds per group. The P-values between groups were determined by one-way ANOVA followed by Tukey's multiple comparisons *post-hoc* test. Only significant comparisons are indicated in the figures.

#### Splenic lymphocyte subset frequencies in the recall response

Flow cytometry was used to assess changes in the frequency of specific lymphocyte subsets in splenocytes in a recall response study. At DPC4, groups 4 and 5 birds had a significantly higher B-cell population compared to other groups (Figure 6Aa). At DPC10, a significantly higher frequency of B-cells was found in group 2 compared to group 4. An increased B-cell population in groups 5 and 6 was comparable but not significant compared to other groups (Figure 6Ab).

**Figure 6:**
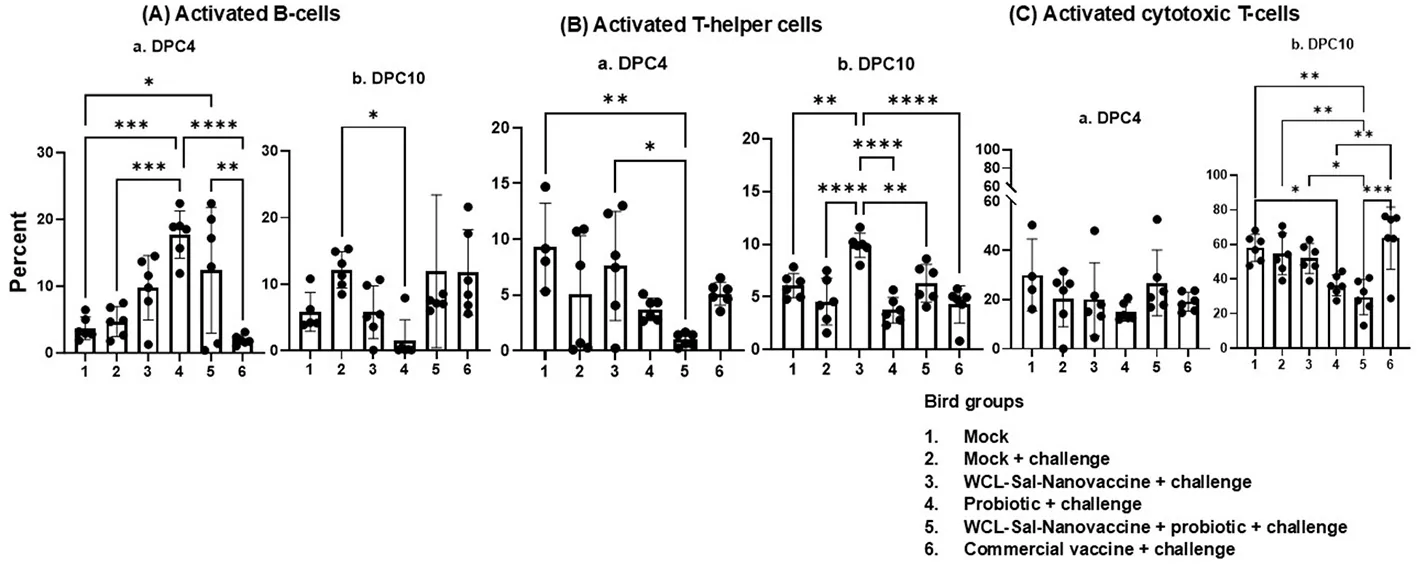
Frequency of activated immune cells in the splenocytes of birds: **(A)** B-cells; **(B)** T-helper cells; **(C)** Cytotoxic T cells. Cells isolated at DPC4 and DPC10 were restimulated with OMP+FLA for 48 hr and then subjected to flow cytometry analysis. Each bar represents the mean ± SEM from 6 birds per group. The *P-*values between groups were determined by one-way ANOVA followed by Tukey's multiple comparisons *post-hoc* test. Only significant comparisons are indicated in the figures.

At DPC4, group 5 had a significantly lower frequency of T-helper cells than groups 1 and 3 (Figure 6Ba). At DPC10, a significantly higher frequency of T-helper cells was observed in group 3 compared to all other groups (Figure 6Bb). At DPC4, no differences were observed in the frequency of cytotoxic T-cells across all experimental groups (Figure 6Ca). However, at DPC10, a significantly lower frequency of cytotoxic T-cells was observed in groups 4 and 5 compared to other groups (Figure 6Cb). TCRγδ cell frequency was also assessed, but there was no difference among the vaccinated groups (data not shown).

### Reduction in SE colonization

All experimental groups except the mock control group were challenged orally with SE 5 × 10^7^ CFU/bird. Bacterial enumeration in the cecal content was performed to determine the challenge SE load at DPC4 and DPC10. At DPC4, birds in groups 2 and 6 had the highest bacterial load with 5.29 log_10_ and 5.71 log_10_ CFU/g, respectively. Notably, groups 4 (probiotic only) and 5 (probiotic combined with WCL-*Salmonella* nanovaccine) showed the greatest reductions in bacterial load with 4.37 log_10_ and 4.47 log_10_ CFU/g of cecal content, respectively, which were statistically significant compared to the commercial vaccine group. These findings indicated that the probiotic-alone helped in reducing the SE colonization to the same extent as the nanovaccine (Figure 7a). At DPC10, groups 3, 4, and 6 showed a numerical reduction in the challenge bacterial load by approximately 1 log_10_ compared to group 2 (challenge) (Figure 7b). Interestingly, group 5 (probiotic combined with WCL-*Salmonella* nanovaccine) showed no further appreciable reduction in bacterial load at DPC10 compared with the levels observed at DPC4 (Figures 7a, b).

**Figure 7:**
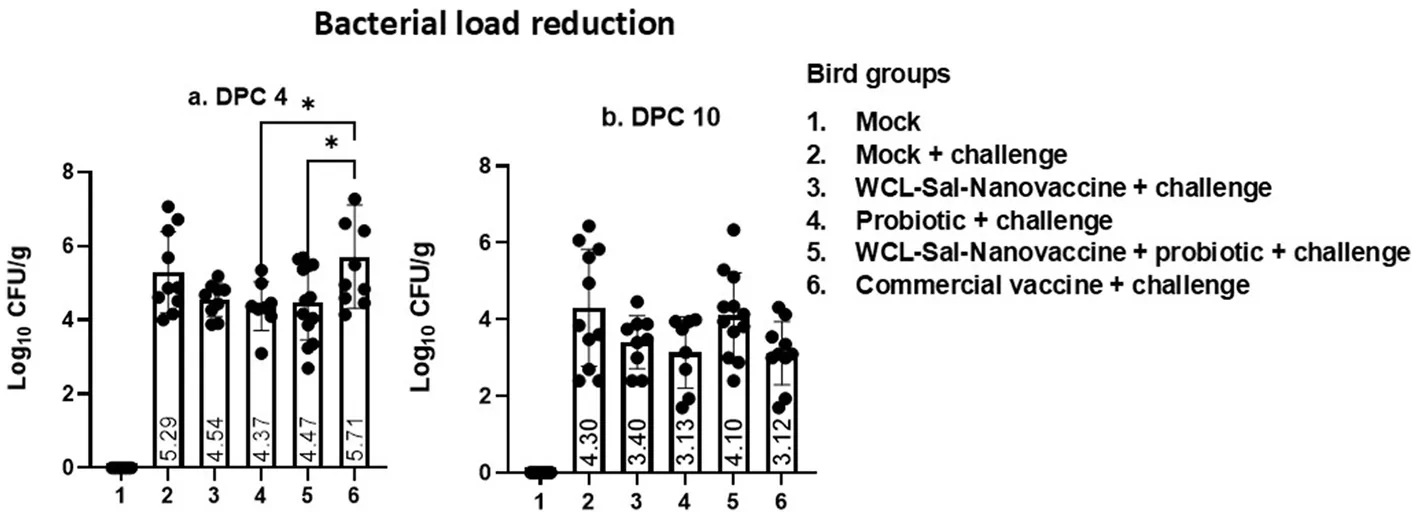
Challenge *Salmonella* Enteritidis load in the cecum of birds. The bacterial enumeration in the cecum was performed at post-challenge days 4 and 10. The cecal content was diluted and plated on XLT4 agar plates supplemented with 20 μg/ml of Nalidixic acid and Novobiocin. Each bar represents the mean ± SEM of log_10_ CFU/g from 8–13 birds per treatment group. Only significant comparisons are indicated in the figures.

### Alterations in cecal microbiota

#### Alpha-diversity differences across treatment groups

Alpha diversity was assessed using multiple indices (Observed, Chao1, ACE, Shannon, Simpson, InvSimpson, Fisher, Pielou, Coverage). Global Kruskal-Wallis tests indicated significant differences among groups for most richness- and diversity-related indices (Observed, Chao1, ACE, Simpson, InvSimpson, Fisher, Pielou; all *p* < 0.05), while Shannon diversity and Coverage did not differ significantly (*p* > 0.05). Dunn's *post hoc* test with Benjamini-Hochberg correction identified a significant difference in community evenness (Pielou) between the control and vaccine+probiotic+SE groups (*p*.adj = 0.039); no other pairwise comparisons remained significant after correction (Figure 8).

**Figure 8:**
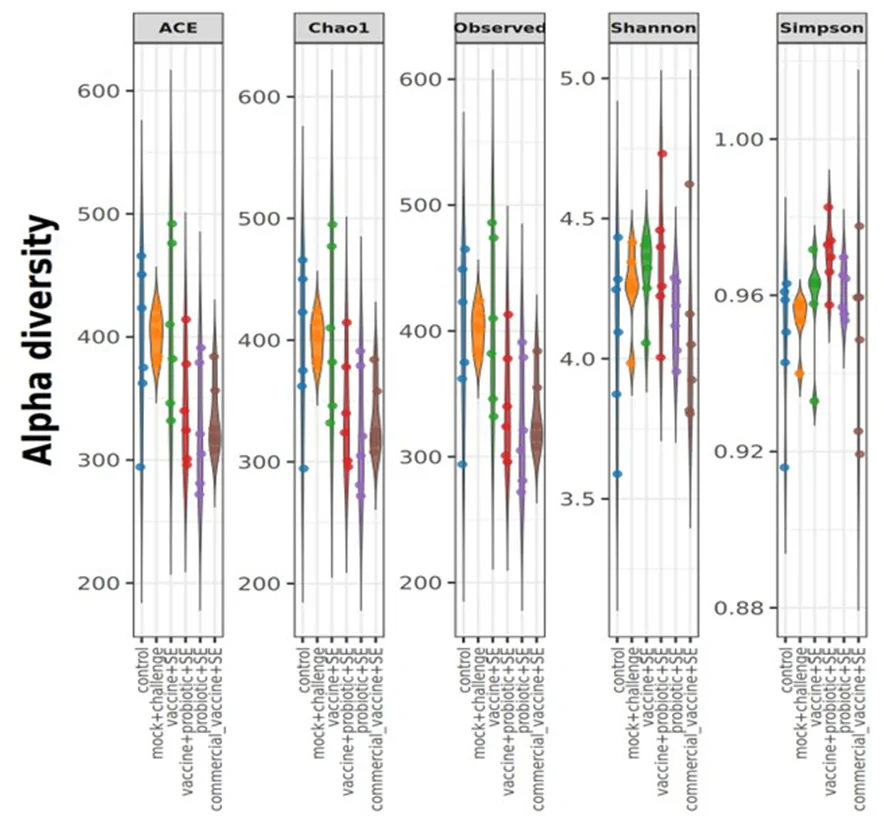
Alpha diversity indices (ACE, Chao1, Observed ASVs, Shannon and Simpson) are shown as violin plots with jittered points representing individual samples. Group differences were assessed using the Kruskal-Wallis test with Dunn's *post hoc* test (Benjamini-Hochberg-adjusted). Significant global differences among groups were detected for ACE, Chao1, Observed ASVs, but not for Shannon diversity (*p* = 0.17); pairwise comparisons remained significant after correction only for Pielou evenness between the control and vaccine+probiotic+SE groups (*p*.adj = 0.039).

#### Beta diversity: distinct community structures driven by probiotic supplementation

Beta-diversity analysis based on Bray–Curtis dissimilarities revealed treatment-dependent clustering of samples in Principal coordinates analysis (Figure 9). PERMANOVA confirmed significant differences in community composition among groups (*R*^2^ = 0.378, *p* = 0.001). PERMDISP was also significant (*F* = 3.676, *p* = 0.006), indicating heterogeneity in within-group dispersion among treatment groups. Pairwise PERMANOVA indicated that several treatment groups (e.g., control vs. vaccine+probiotic+SE and mock+challenge vs. commercial vaccine+SE) differed significantly (*p* < 0.01). Pairwise comparisons showed that the probiotic-only (Group 4) and probiotic+nanovaccine (Group 5) groups differed significantly from the commercial vaccine group (Group 6; *p* = 0.016), whereas Group 4 and Group 5 did not differ from each other (*p* = 0.355). Although PERMDISP detected some dispersion effects (*p* = 0.006), the consistent PERMANOVA supports a meaningful compositional divergence between Groups 5 and 6.

**Figure 9:**
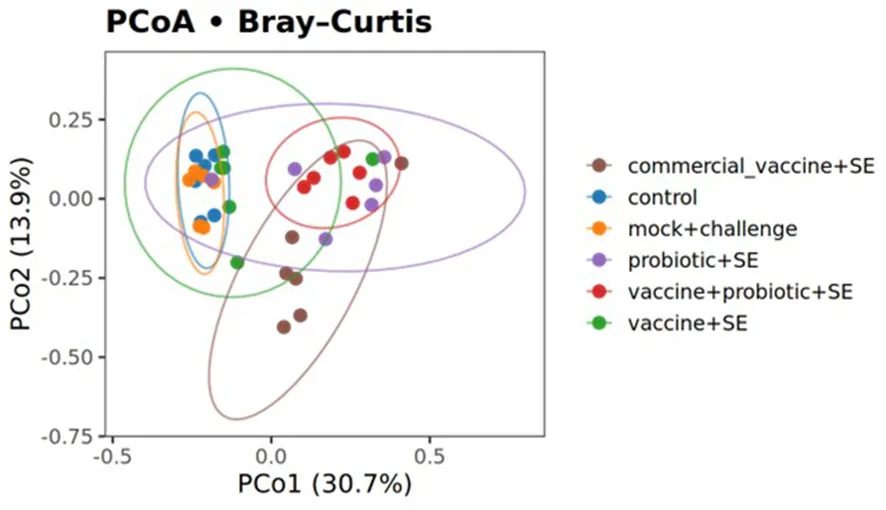
Principal coordinates analysis (PCoA) of Bray–Curtis dissimilarities of cecal microbiota by treatment group. Ellipses represent 95% confidence intervals around group centroids. Although the ellipses show partial overlap across groups, PERMANOVA identified a significant, moderate-effect-size difference in overall community composition (*R*^2^ = 0.378, *p* = 0.001, 999 permutations). PERMDISP indicated significant heterogeneity in within-group dispersion (*F* = 3.676, *p* = 0.006), which may contribute to some of the apparent overlap and should be considered when interpreting group separation visually. Probiotic+vaccine (red) clustered separately from the commercial vaccine group (brown), while probiotic (purple) and probiotic+vaccine (red) showed greater overlap, consistent with the pairwise PERMANOVA result that these two groups did not differ significantly from each other (*p* = 0.355) despite each differing significantly from the commercial vaccine group (*p* = 0.016).

#### Taxonomic composition: probiotic-nanovaccine combination enriches SCFA-producing *Clostridiales* and other immunomodulatory taxa

The probiotic-only group (Group 4) enriched several beneficial taxa, including putative short-chain fatty acid producers, such as *Blautia* and *Butyricicoccus*. These effects persisted in the probiotic+vaccine group (Group 5), along with additional enrichment of immunomodulatory genera, including Christensenellaceae R-7_group and *Oscillibacter*. In contrast, the commercial vaccine group (Group 6) showed a higher relative abundance of *Escherichia/Shigella* and a reduced abundance of *Lactobacillus*, reflecting a less beneficial microbial profile (Figures 10A, B).

**Figure 10:**
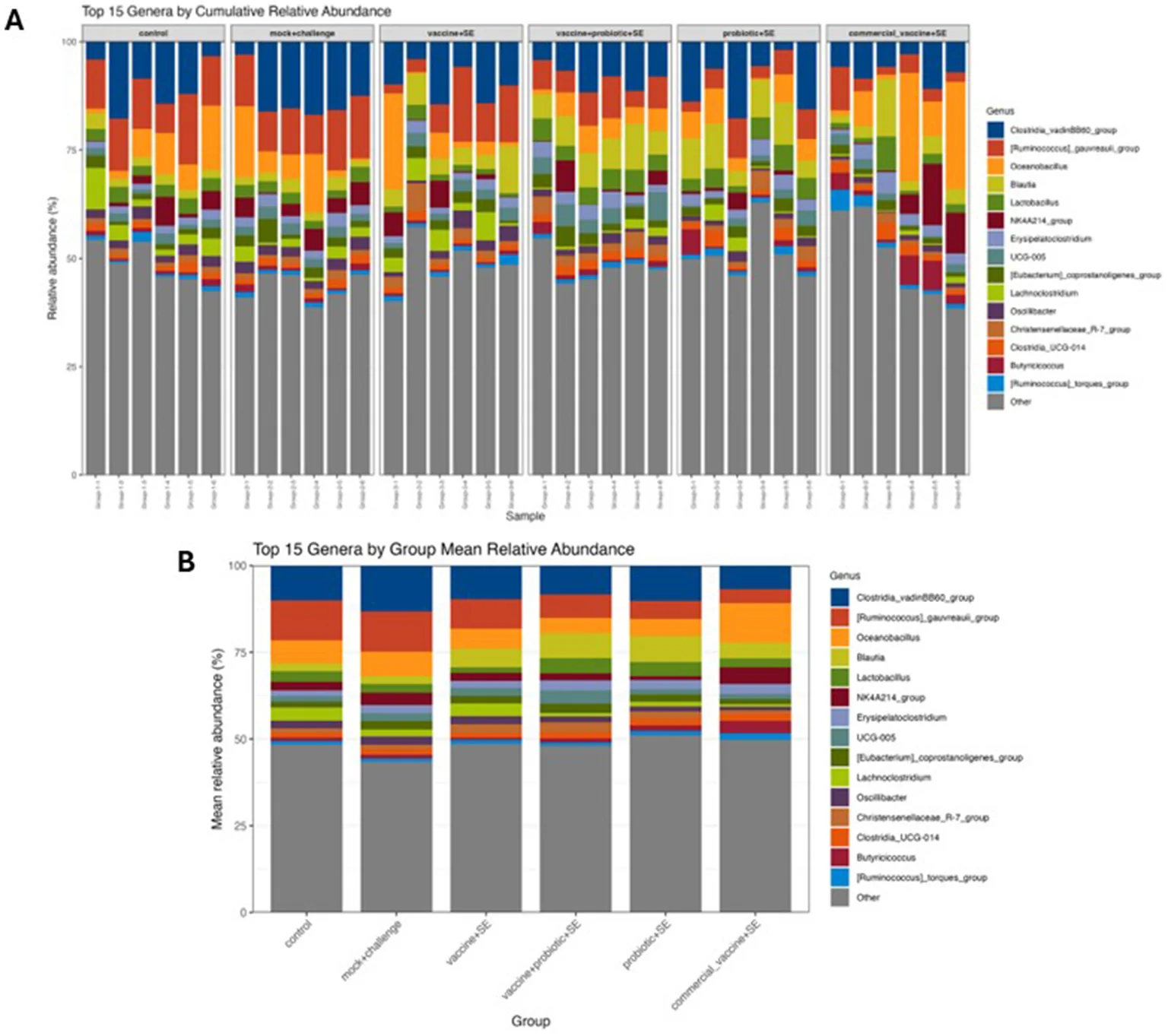
Relative abundance of the top 15 bacterial genera in the cecal microbiota across treatment groups. **(A)** Stacked bar plots showing the cumulative relative abundance of the 15 most abundant bacterial genera in individual samples from each treatment group: control (uninfected), mock+challenge (SE only), vaccine+SE, vaccine+probiotic+SE, probiotic+SE, and commercial_vaccine+SE. The cecal microbiota was predominantly composed of members of the order Clostridiales, including Clostridia_vadinBB60_group, [Ruminococcus]_gauvreauii_group, *Blautia*, and *Oscillibacter*, along with *Lactobacillus* and Christensenellaceae_R-7_group. **(B)** Group means relative abundance profiles of the top 15 bacterial genera across treatment groups. Probiotic-supplemented groups, particularly the vaccine+probiotic+SE and probiotic+SE groups, exhibited increased relative abundance of beneficial Clostridiales-associated genera, including *Blautia, Oscillibacter*, and Christensenellaceae_R-7_group, compared with the commercial vaccine group.

### Differential abundance analyses highlight synergistic signatures

LEfSe identified 46 taxa with significant differential abundance across groups (LDA > 2, *p* < 0.05) (Figure 11). The probiotic-only (Group 4) and probiotic+nanovaccine+SE (Group 5) groups were characterized by biomarkers associated with barrier integrity and SCFA production, including Christensenellaceae R-7_group, *Blautia, Intestinimonas, Butyricicoccus*, and Lachnospiraceae NK4A136_group. These lineages are recognized as butyrogenic *Clostridiales* members. The commercial vaccine group (Group 6) exhibited enrichment of carbohydrate-fermenting anaerobes like *Clostridines, Sellimonas, Dickeyea, RF39, Anaerostipes*, and *UBA1819*, distinct from commercial vaccination.

**Figure 11:**
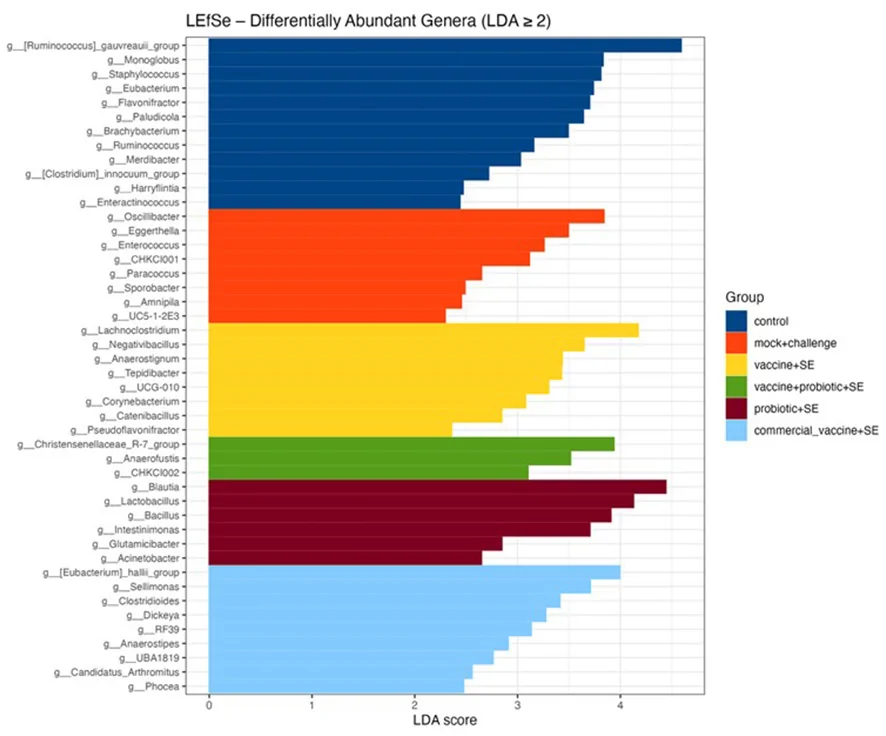
Differentially abundant genera across treatment groups identified by LEfSe analysis. Linear discriminant analysis (LDA) effect size (LEfSe) identified bacterial genera that were differentially enriched across treatment groups (LDA scores > 2). Bars represent taxa enriched in each group, color-coded by treatment.

In contrast, the mock+SE group (Group 2) and the nanovaccine+SE group (Group 3) were characterized by enrichment of *Lachnoclostridium, Negativibacillus, Anaerostignum*, and *Teplitzbacteria*, taxa often associated with dysbiosis. The uninfected control group (Group 1) exhibited a higher abundance of commensal *[Ruminococcus]_gauvreauii_group, Monoglobus*, and *Staphylococcus*, indicating a stable baseline microbiota.

Using MaAsLin2, we detected group-associated differences in genus-level relative abundance compared with controls (*q* < 0.25). The most consistent negative associations were observed for *Flavonifractor, Corynebacterium, Enteractinococcus, Amnipila*, and *Enterococcus*, particularly in probiotic-containing or commercial vaccine groups (Figure 12). Positive associations were observed among selected taxa, including *Anaerostignum, Blautia, Sellimonas*, and *Acinetobacter*, although these patterns varied across treatment groups. These findings indicate that the treatment groups exhibited distinct microbial signatures relative to the control group. Where LEfSe and MaAsLin2 produced concordant findings, results were interpreted as higher-confidence associations. Taxa detected by both LEfSe and MaAsLin2, including *Blautia, Anaerostignum*, and *Sellimonas*, were considered the strongest treatment-associated candidates.

**Figure 12:**
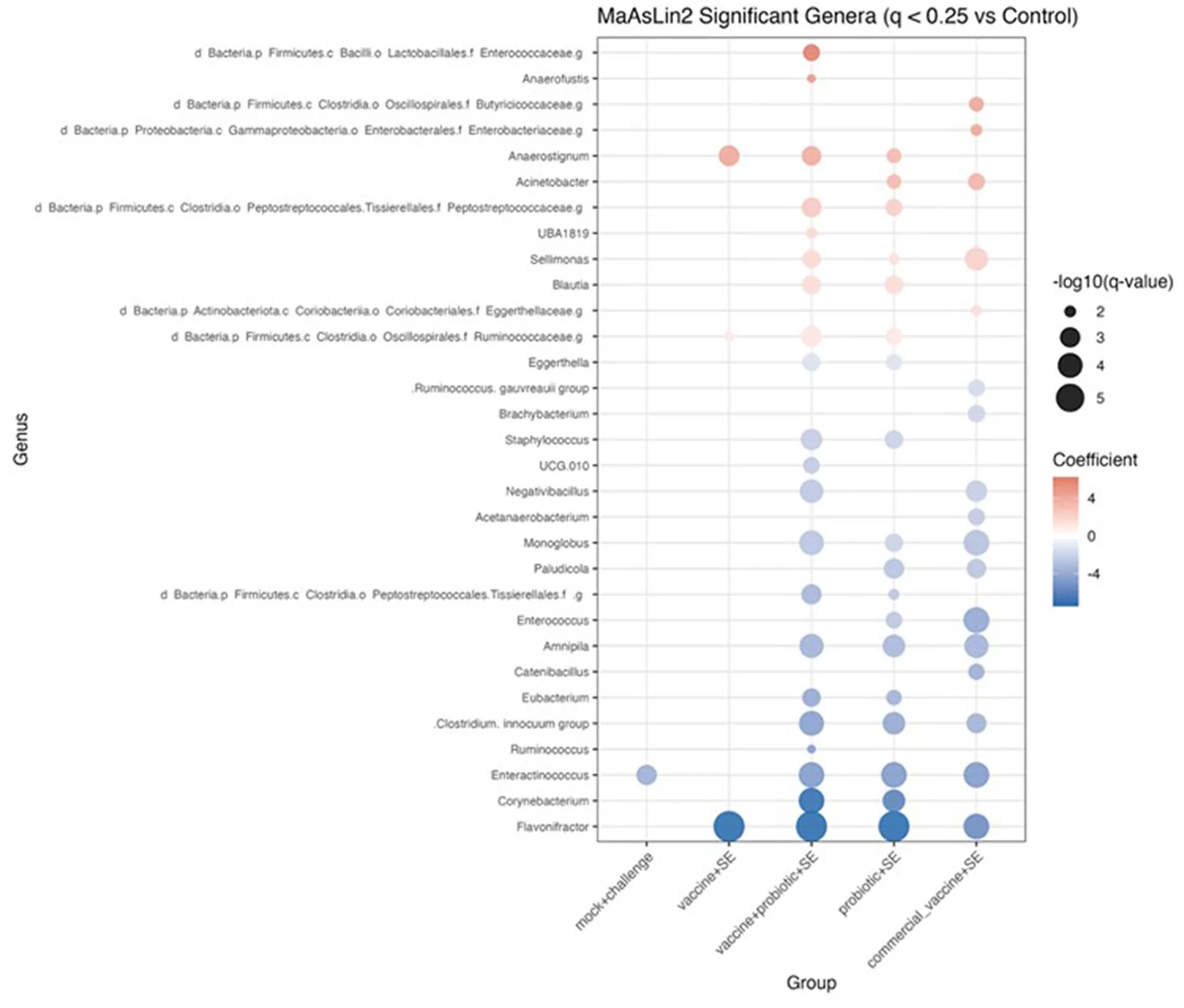
MaAsLin2 analysis of treatment-associated genera. Bubble plot showing genus-level taxa significantly associated with treatment groups relative to the control group using MaAsLin2 (*q* < 0.25). Point color indicates the regression coefficient: red for positive associations and blue for negative associations compared with controls. Point size represents significance as log_10_(q-value). Only significant genus-level associations are shown.

A family-level heatmap (Figure 13) of log-transformed relative abundances demonstrated that probiotic supplementation, either alone or combined with the nanovaccine, maintained a Clostridiales-rich cecal community dominated by SCFA-producing taxa. The probiotic-only (Group 4) and probiotic+nanovaccine (Group 5) groups exhibited higher abundance of Lachnospiraceae, Christensenellaceae, and Ruminococcaceae, consistent with the LEfSe analysis.

**Figure 13:**
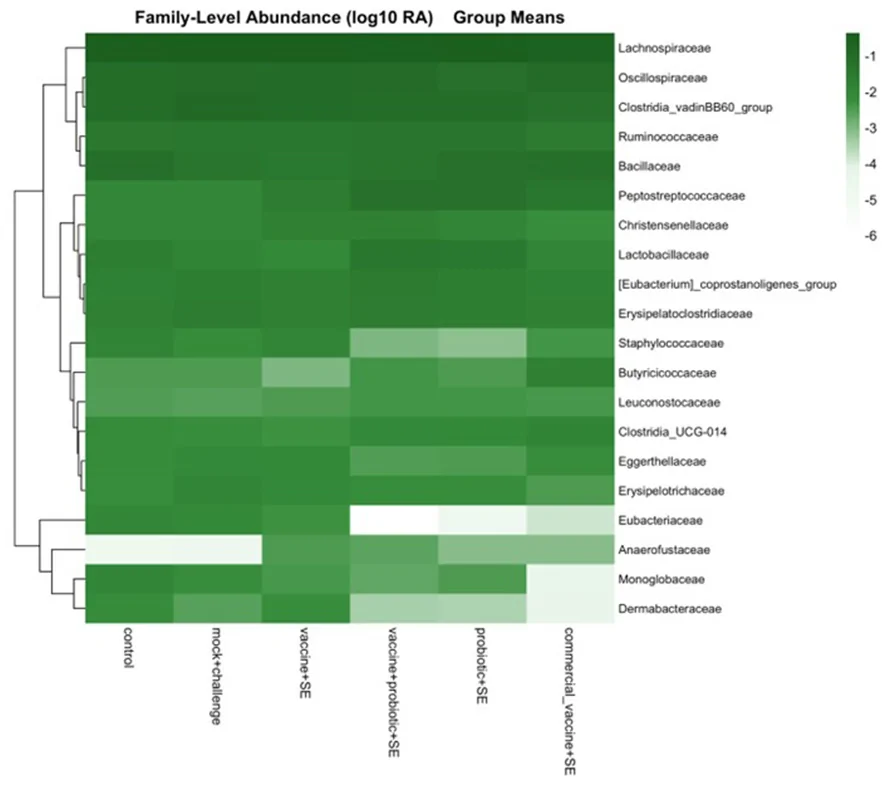
Heatmap of the top 20 most abundant bacterial families in the cecal microbiota of broiler chickens across treatment groups. Relative abundance values were log_10_-transformed and averaged within each treatment group prior to visualization. Darker green indicates higher relative abundance, whereas lighter shades indicate lower relative abundance. Hierarchical clustering showed that probiotic-supplemented groups, particularly the probiotic+vaccine+SE group, were enriched for families within Clostridiales, including Lachnospiraceae, Christensenellaceae, and Ruminococcaceae, compared with the commercial vaccine group.

## Discussion

This study provides compelling evidence for the adjuvant potential of probiotic *L. acidophilus* in enhancing the protective efficacy of an orally administered mannose-conjugated chitosan nanoparticle-based *Salmonella* subunit vaccine supplemented with *M. smegmatis* whole cell lysate (WCL) against SE infection in broilers. Though the difference in bacterial clearance following challenge was not substantial between the vaccine and probiotics alone groups, integrating immunological evaluations with microbiome analysis demonstrated that this dual-adjuvant approach not only induced robust mucosal and systemic immune responses, but also modulated the gut microbiota in a manner that supports reduced pathogen colonization. Therefore, this strategy offers a promising alternative to conventional vaccines in antibiotic-free poultry production.

The physicochemical characterization of the nanovaccine reaffirmed its suitability for oral delivery with a positive zeta potential facilitating mucoadhesion, and particle sizes optimal for uptake by M-cells and APCs in the GALT (Oyewumi et al., 2010). These properties align with previous optimizations in our laboratory, where mannose-chitosan NPs enhanced antigen presentation and cross-protection of SE nanovaccine against the *S*. Typhimurium challenge (Dolatyabi et al., 2024). The adjuvant WCL is devoid of toxic mycobacterial components, and it contributes to innate immune activation through ligands like MDP and TDM, which engage pattern recognition receptors on APCs. Specifically, MDP is recognized by the cytosolic receptor NOD2, while TDM signals through the C-type lectin receptor Mincle (Macrophage-inducible C-type lectin), both activate the NF-κB–driven inflammatory pathway that enhances antigen presentation and downstream adaptive immune responses (Hansen et al., 2014; Binjawadagi et al., 2014).

In Phase 1, probiotic administration modulated Th2 (IL-4), Th17 (IL-17), and Th1 (IFN-γ) cytokines — pathways central to mucosal antibody responses, neutrophil recruitment, and intracellular pathogen control, respectively, consistent with priming toward anti-Salmonella immunity. This early modulation is consistent with the role of probiotics in establishing a healthy microbiota in commercially hatched chicks, where natural microbial transfer from hens is lacking (Rychlik, 2020). These directional Th1/Th17-associated changes are consistent with priming toward responses relevant to combating enteric pathogens like *Salmonella* (Chi et al., 2024).

Phase 2 results highlighted the contribution of the probiotic-nanovaccine combination to mucosal immunity. Although pre-challenge systemic IgY and early sIgA were not significantly increased, antigen-specific mucosal sIgA differences emerged post-challenge at the site of colonization (anti-OMP sIgA in small intestinal wash, group 5 vs. commercial vaccine). Protection in this model therefore reflects combined mucosal, cellular, and microbiome-mediated mechanisms rather than serum antibody titer alone (Sayegh et al., 2000). Secretory IgA plays a pivotal role in preventing bacterial adhesion and colonization, and the higher levels of specific sIgA responses in the intestines and bile of the WCL-Sal-Nanovaccine + Probiotic group at DPC10 compared to a commercial live vaccine indicate superior mucosal protection. This aligns with the recognized advantages of subunit vaccines over live vaccines, particularly the latter may persist in the environment.

Cytokine profiling in cecal tonsils revealed a dynamic immune landscape characterized by prevention of excessive inflammation (Quast et al., 2013). These patterns support balanced immunity, potentially mitigating immunopathology while sustaining protection Th1/Th17 dominance at DPC4 that likely contributed to rapid pathogen clearance, followed by a transition toward IL-10-mediated regulation at DPC10 to prevent excessive inflammation (Huang, 2021). Non-significant directional changes in cytokine expression, in both Phase 1 and Phase 2 trials, are interpreted as exploratory rather than confirmatory. These patterns support balanced immunity, potentially mitigating immunopathology while sustaining protection. Flow cytometry data further corroborate adaptive responses, with increased frequencies of activated B cells, T-helper cells and cytotoxic T-cells in the spleen. Although B-cell frequencies were elevated in the probiotic-nanovaccine groups, this did not translate into significantly higher systemic IgY. This dissociation likely reflects that the increased B-cells represent early activation and mucosal-oriented responses rather than terminal differentiation into IgY-secreting plasma cells. Oral mucosal immunization preferentially drives local sIgA over systemic IgY, and the sampling window (DPC4 and DPC10) may have preceded peak antibody class-switching and affinity maturation. The probiotic's contribution likely stems from its immunomodulatory effects, including enhanced dendritic cell maturation and IgA class-switching (Erickson and Hubbard, 2000). The probiotic lactobacilli enhance the maturation of the bursa of Fabricius and increases the expression of TGF-β that is responsible for isotype switching of IgM to IgA antibodies (Sharma et al., 2024). The lower T-helper and cytotoxic T-cell frequencies observed at DPC10 in the probiotic and probiotic-nanovaccine groups are consistent with effector contraction and a regulatory transition (concurrent with elevated IL-10 and the lowest cecal SE burden), rather than diminished immunity, whereas the persistently higher cytotoxic T-cell frequency in the commercial live-vaccine group reflects ongoing stimulation by a replicating intracellular organism.

An observed reduction in cecal SE load and associated immune responses supports the protective efficacy of this mucosal vaccination approach, consistent with cross-protection studies using conserved antigens like OMP and FLA (Renu et al., 2020). On cecal colonization, however, probiotic supplementation alone and the probiotic-nanovaccine combination produced comparable reductions (4.37 vs. 4.47 log_10_), and we do not interpret the combination as providing an additive reduction in bacterial load. The distinct contribution of the nanovaccine was instead evident in antigen-specific mucosal immunity (anti-OMP sIgA in the small intestine, group 5 vs. group 3 and the commercial vaccine), sustained B-cell recall, and additional immunomodulatory microbiome shifts (Dolatyabi et al., 2024). The absence of further colonization reduction in Group 5 between DPC4 and DPC10 likely reflects that bacterial clearance had already reached a plateau by DPC4, when antigen-specific and probiotic-driven mechanisms were maximally active. The early reduction coincided with peak Th1/Th17 responses, after which the immune profile transitioned toward IL-10-mediated regulation (as reflected in the DPC10 cytokine data), prioritizing resolution of inflammation over continued bacterial killing. In addition, the residual bacterial load at DPC10 may represent a colonization-resistant population in a niche less accessible to mucosal effectors, such that the rate of clearance slows once the readily cleared fraction has been eliminated.

These outcomes emphasize the value of vaccination via the mucosal route that mimics natural infection, stimulating GALT and reducing fecal shedding, which is a key factor in zoonotic transmission.

We found that harvested OMP and flagella contain endotoxin and its levels were 0.000199 EU/μg and 0.000289 EU/μg, respectively, which corresponds to much lower levels of endotoxin present in each dose of our nanovaccine formulation as per the acceptable levels of endotoxin in each vaccine dose (Brito and Singh, 2011). Therefore, it is unlikely that vaccine-specific responses that we detected were from endotoxin-mediated immune activation.

Microbiome analysis at DPC10 provided novel insights into how the mucosally-administered vaccines and a probiotic modulate the microbial ecosystem in *Salmonella*-infected birds. The lack of significant changes in alpha diversity and significant global changes reported for multiple indices suggest that the vaccine and a probiotic formulation tested in this study did not disrupt overall microbial richness, a desirable trait for maintaining gut health (Shehata et al., 2022). However, beta diversity revealed distinct community structures, with probiotic+nanovaccine groups clustering separately from the commercial vaccine. The enrichment of butyrogenic *Clostridiales*, such as *Blautia, Butyricicoccus*, Christensenellaceae R-7_group, and *Oscillibacter*, in probiotic-treated birds is particularly noteworthy. These bacteria produce butyrate, which supports epithelial barrier integrity and enhances mucin production, in addition to their anti-inflammatory effects. Biomarkers identified by LEfSe further highlighted synergistic effects, with probiotic-vaccine combinations fostering immunomodulatory genera absent in commercial vaccine profiles. These shifts may underlie the observed immune enhancements, as a resilient microbiota supports vaccine responsiveness. The distinct microbiota shift in the commercial live-vaccine group likely reflects the nature of a replicating live-attenuated organism, which transiently colonizes and perturbs the gut environment, favoring facultative anaerobes such as *Escherichia/Shigella* and reducing beneficial Lactobacillus. In contrast, the non-replicating subunit nanovaccine and probiotic preserved a Clostridiales-rich, SCFA-producing community.

In summary, the probiotic-nanovaccine combination in orally vaccinated broilers reduced cecal SE load by 1.2 log_10_, performing slightly better than a commercial live *Salmonella* vaccine. Immunologically, this vaccination strategy was associated with substantially increased OMP-specific sIgA production in the intestines and a higher frequency of activated B-cells compared to the commercial live vaccine. Therefore, our data strongly support the hypothesis that *L. acidophilus* potentiates the nanovaccine-induced mucosal immunity and contributes to reducing SE colonization through integrated immunity, while favorably modulating the gut microbiome. We also note that PERMDISP indicated significant heterogeneity in within-group dispersion (*F* = 3.676, *p* = 0.006) because PERMANOVA is sensitive to differences in group dispersion as well as differences in centroid location, the significant PERMANOVA result (*R*^2^ = 0.378, *p* = 0.001) may partially reflect unequal dispersion among treatment groups rather than centroid separation alone, and should be interpreted with this caveat in mind. This approach addresses the limitations of current strategies, including concerns about antimicrobial resistance and the need for scalable stress-free vaccination strategy in broilers. However, long-term studies could assess vaccine durability and microbiome stability, and we acknowledge that fully establishing adjuvanticity on the colonization endpoint would require demonstrating a nanovaccine-dependent reduction beyond probiotic alone, which the current data do not show; we note this explicitly as a limitation and a direction for future work. Future research should explore multi-strain probiotics, additional vaccine boosters, and metagenomic analyses of the microbiota to gain functional insights into the altered microbiota for improving this promising nanovaccine-probiotic approach against *Salmonella* foodborne illness in broilers.

## Conclusions

In conclusion, our immunological responses data supports the potent adjuvant effect of the probiotic *L. acidophilus* in an orally delivered *Salmonella* subunit nanovaccine, primarily through the induction of mucosal immunity in broilers. Microbiome analysis further validated the efficacy of this immunization strategy, wherein the probiotic-nanovaccine combination induced immune priming in the GALT alongside vaccine-mediated antigen-specific immune responses. These findings suggest integrating probiotics with a nanoparticle-based subunit vaccine represents a novel strategy to enhance protective mucosal immunity against *Salmonella* in poultry. By achieving protection comparable to a commercial live Salmonella vaccine, this probiotic-nanovaccine combination strategy supports the shift toward antibiotic-free poultry production, lowering the risk of *Salmonella* transmission while avoiding issues related to antimicrobial resistance.

## Data Availability

The data analyzed in this study is available in the NCBI repository (https://www.ncbi.nlm.nih.gov/bioproject) under BioProject accession PRJNA1469281.

## References

[B1] Apaka R. R. (2024). How do adjuvants enhance immune responses? eLife 13:101259. doi: 10.7554/eLife.101259 39136115 PMC11321759

[B2] Binjawadagi B. Dwivedi A. Renu S. Renukaradhya G. J. (2014). An innovative approach to induce cross-protective immunity against porcine reproductive and respiratory syndrome virus in the lungs of pigs through adjuvanted nanotechnology-based vaccination. Int. J. Nanomedicine 9, 1519–1535. doi: 10.2147/IJN.S59924 24711701 PMC3969340

[B3] Brito L. A. Singh M. (2011). Acceptable levels of endotoxin in vaccine formulations during preclinical research. J. Pharm. Sci. 100, 34–37. doi: 10.1002/jps.22267 20575063

[B4] Chagas D. B. Santos F. D. S. de Oliveira N. R. Bohn T. L. O. Dellagostin O. A. (2024). Recombinant live-attenuated salmonella vaccine for veterinary use. Vaccines 12:1319. doi: 10.3390/vaccines12121319 39771981 PMC11680399

[B5] Chi H. Wu L. Wang G. Zhang X. Li Y. (2024). Principles and therapeutic applications of adaptive immunity. Cell 187 2052–2078. doi: 10.1016/j.cell.03, 037 PMC11177542 38670065

[B6] Diaz Carrasco J. M. Casanova N. A. Casanova A. Fernández Miyakawa M. E. (2019). Microbiota, gut health and chicken productivity: what is the connection? Microorganisms 7:374. doi: 10.3390/microorganisms7100374 31547108 PMC6843312

[B7] Dolatyabi S. Renu S. Schrock J. Renukaradhya G. J. (2024). Chitosan-nanoparticle-based oral Salmonella Enteritidis subunit vaccine elicits cross-protection against Salmonella Typhimurium in broilers. Poult. Sci.103:103569. doi: 10.1016/j.psj.2024 103569 38447310 10.1016/j.psj.2024.103569 PMC11067733

[B8] El-Shall N. A. Abd El-Hack M. E. Alagawany M. Taha A. E. Swelum A. A. El-Saadony M. T. . (2019). The simultaneous administration of a probiotic or prebiotic with live salmonella vaccine improves growth performance and reduces fecal shedding of the bacterium in salmonella-challenged broilers. Animals 10:70. doi: 10.3390/ani10010070 31906020 PMC7023486

[B9] Erickson K. L. Hubbard N. E. (2000). Probiotic immunomodulation in health and disease12. J. Nutr. 130, 403S–409S. doi: 10.1093/jn/130.2.403S 10721915

[B10] European Food Safety Authority European Centre for Disease Prevention and Control (2023). The european union one health 2022 zoonoses report. EFSA J. 21:e8442. doi: 10.2903/j.efsa.2023.8442 38089471 PMC10714251

[B11] Ford L. Glass J. Hall G. Kirk M. D. Veitch M. Lalor K. . (2019). Cost of salmonella infections in Australia, 2015. J. Food Prot. 82, 1607–1614. doi: 10.4315/0362-028X.JFP-19-105 31436484

[B12] Guo F. Zhang J. Li Y. Wang X. Chen H. Liu Z. . (2023). Immunological effects of the PE/PPE family proteins of Mycobacterium tuberculosis and related vaccines. Front. Immunol. 14:1255920. doi: 10.3389/fimmu.2023.1255920 37841250 PMC10569470

[B13] Han Y. Renu S. Patil V. Schrock J. Feliciano-Ruiz N. Selvaraj R. . (2020). Mannose-modified chitosan-nanoparticle-based salmonella subunit oralvaccine-induced immune response and efficacy in a challenge trial in broilers. Vaccines 8:299. doi: 10.3390/vaccines8020299 32545295 PMC7349978

[B14] Hansen J. M. Golchin S. A. Veyrier F. J. Domenech P. Boneca I. G. Azad A. K. . (2014). N-glycolylated peptidoglycan contributes to the immunogenicity but not pathogenicity of Mycobacterium tuberculosis. J. Infect. Dis. 209, 1045–1054. doi: 10.1093/infdis/jit622 24265438

[B15] Huang F. C. (2021). The interleukins orchestrate mucosal immune responses to salmonella infection in the intestine. Cells 10:3492. doi: 10.3390/cells10123492 34943999 PMC8700606

[B16] Kozich J. J. Westcott S. L. Baxter N. T. Highlander S. K. Schloss P. D. (2013). Development of a dual-index sequencing strategy and curation pipeline for analyzing amplicon sequence data on the MiSeq Illumina sequencing platform. Appl. Environ. Microbiol. 79, 5112–5120. 23793624 10.1128/AEM.01043-13 PMC3753973

[B17] Lépine A. F. P. de Wit N. Oosterink E. Wichers H. Mes J. de Vos P. (2018). Lactobacillus acidophilus attenuates salmonella-induced stress of epithelial cells by modulating tight-junction genes and cytokine responses. Front. Microbiol. 9:1439. doi: 10.3389/fmicb.2018.01439 30013538 PMC6036613

[B18] Liu Q. Ruan S. Wang X. Li Y. Zhang H. Chen Y. . (2016). Outer membrane vesicles derived from Salmonella Typhimurium mutants with truncated LPS induce cross-protective immune responses. Int. J. Med. Microbiol. 306 697–706. doi: 10.1016/j.ijmm.08, 004. PMC5206754 27578609

[B19] Lone A. Renu S. Renukaradhya G. J. (2022). Bacteriocinogenic probiotics as an integrated alternative to antibiotics in chicken production—why and how? Crit. Rev. Food Sci. Nutr. 62, 8744–8760. doi: 10.1080/10408398.2021.1932722 34060404

[B20] MacLennan C. A. Martin L. B. Lee S. J. (2014). Vaccines against invasive Salmonella disease: current status and future directions. Hum. Vaccin. Immunother. 10, 1478–1493. doi: 10.4161/hv.29054 24804797 PMC4185946

[B21] Majowicz S. E. Musto J. Scallan E. Angulo F. J. Kirk M. D. O'Brien S. J. . (2010). The global burden of nontyphoidal Salmonella gastroenteritis. Clin. Infect. Dis. 50, 882–889. doi: 10.1086/650733 20158401

[B22] Nazir J. Hussain A. Mubeen S. Aslam M. Khan S. Khan M. . (2025). Combatting Salmonella: a focus on antimicrobial resistance and the need for effective vaccination. BMC Infect. Dis. 25:84. doi: 10.1186/s12879-025-10478-5 39833704 PMC11744889

[B23] Oyewumi M. O. Kumar A. Cui Z. (2010). Nano-microparticles as immune adjuvants: correlating particle sizes and the resultant immune responses. Expert Rev. Vaccines 9, 1095–1107. doi: 10.1586/erv.10.89 20822351 PMC2963573

[B24] Quast C. Pruesse E. Yilmaz P. Gerken J. Schweer T. Yarza P. . (2013). The SILVA ribosomal RNA gene database project: improved data processing and web-based tools. Nucleic Acids Res. 41, D590–D596. doi: 10.1093/nar/gks1219 23193283 PMC3531112

[B25] Renu S. Feliciano-Ruiz N. Patil V. Schrock J. Han Y. Ramesh A. . (2021). Immunity and protective efficacy of mannose conjugated chitosan-based influenza nanovaccine in maternal antibody positive pigs. Front. Immunol. 12:584299. doi: 10.3389/fimmu.2021.584299 33746943 PMC7969509

[B26] Renu S. Markazi A. D. Dhakal S. Lakshmanappa Y. S. Gourapura S. R. Shanmugasundaram R. . (2018). Surface engineered polyanhydride-based oral Salmonella subunit nanovaccine for poultry. Int. J. Nanomedicine 13, 8195–8215. doi: 10.2147/IJN.S185588 30555234 PMC6280892

[B27] Renu S. Markazi A. D. Dhakal S. Lakshmanappa Y. S. Shanmugasundaram R. Selvaraj R. K. . (2020). Oral deliverable mucoadhesive chitosan-salmonella subunit nanovaccine for layer chickens. Int. J. Nanomedicine 15, 761–777. doi: 10.2147/IJN.S238445 32099364 PMC7006855

[B28] Renu S. Renukaradhya G. J. (2020). Chitosan nanoparticle based mucosal vaccines delivered against infectious diseases of poultry and pigs. Front. Bioeng. Biotechnol. 8:558349. doi: 10.3389/fbioe.2020.558349 33282847 PMC7691491

[B29] Rychlik I. (2020). Composition and function of chicken gut microbiota. Animals 10:10103. doi: 10.3390/ani10010103 31936291 PMC7022619

[B30] Salman H. H. Irache J. M. Gamazo C. (2009). Immunoadjuvant capacity of flagellin and mannosamine-coated poly(anhydride) nanoparticles in oral vaccination. Vaccine 27 4784–4790. doi: 10.1016/j.vaccine.05, 091 19539576

[B31] Sayegh C. E. Demers M. Cooper M. D. Ratcliffe M. J. H. (2000). The chicken B-cell receptor complex and its role in avian B-cell development. Immunol. Rev. 175, 187–200. doi: 10.1034/j.1600-065X.2000.017507.x 10933603

[B32] Scallan E. Hoekstra R. M. Angulo F. J. Tauxe R. V. Widdowson M. A. Roy S. L. . (2011). Foodborne illness acquired in the United States–major pathogens. Emerging Infect. Dis. 17, 7–15. doi: 10.3201/eid1701.P11101 21192848 PMC3375761

[B33] Shaji S. Suresh A. Renu S. Renukaradhya G. J. (2023). Salmonella infection in poultry: a review on the pathogen and control strategies. Microorganisms 11:2814. doi: 10.3390/microorganisms11112814 38004824 PMC10672927

[B34] Sharma S. Kulkarni R. R. Sharif S. Hassan H. Alizadeh M. Pratt S. . (2024). In ovo feeding of probiotic lactobacilli differentially alters expression of genes involved in the development and immunological maturation of bursa of Fabricius in pre-hatched chicks. Poult. Sci. 103:103237. doi: 10.1016/j.psj.2023.103237 38011819 PMC10801656

[B35] Shehata A. A. El-Sawy A. E. El-Hack M. E. A. Alagawany M. Farag M. R. (2022). Probiotics, prebiotics, and phytogenic substances for optimizing gut health in poultry. Microorganisms 10:395. doi: 10.3390/microorganisms10020395 35208851 PMC8877156

[B36] Suresh R. Shekoni O. Dolatyabi S. Schrock J. Singh M. Renukaradhya G. . (2025). Evaluation of mucosal adjuvants to chitosan-nanoparticle-based oral subunit vaccine for controlling salmonellosis in broilers. Front. Immunol. 16:1509990. doi: 10.3389/fimmu.2025-1509990 39981235 PMC11840259

[B37] Tauxe R. V. (1991). Salmonella: a postmodern pathogen. J. Food Prot. 54, 563–568. doi: 10.4315/0362-028X-54.7.563 31051539

[B38] Vencia W. Iacumin L. Comin A. Cianflone G. Sorrentino A. Losasso G. . (2015). A Salmonella Enterica Subsp. Enterica Serovar Enteritidis Foodborne Outbreak after Consumption of Homemade Lasagne. Ital. J. Food Saf. 4:5127. doi: 10.4081/ijfs.2015.5127 27800418 PMC5076683

[B39] Zhao K. Chen G. Shi X. M. Gao T. T. Li W. Zhao Y. . (2012). Preparation and efficacy of a live newcastle disease virus vaccine encapsulated in chitosan nanoparticles. PLoS One 7:e53314. doi: 10.1371/journal.pone.0053314 23285276 PMC3532065

