# Supplementary material for: Probiotic Lactobacillus acidophilus provides an adjuvant effect to an orally administered chitosan nanoparticle-based Salmonella subunit vaccine in broilers against Salmonella Enteritidis infection
Source: Front Microbiol. 2026 Oct 15;17:1898587. doi: 10.3389/fmicb.2026.1898587 (PMC13717618; doi:10.3389/fmicb.2026.1898587)
Supplement: Supplementary file 1 [file Data_Sheet_1.docx]

**Supplementary Data**

**Table S1:** Antibodies used in the Flow cytometry analysis- Panel 1

| **Specific antibodies** | **Isotype/ fluorochrome** | **Source** |
| --- | --- | --- |
| CD3 AF700 | Mouse IgG1 AF700 | Southern Biotech, Birmingham, AL |
| CD4 AF488 | Mouse IgG1 FITC | Southern Biotech, Birmingham, AL |
| CD8α PE | Mouse IgG1 PE | Southern Biotech, Birmingham, AL |
| CD8 β Biotin | Mouse IgG2 Biotin  Conjugate: SA PE Texas Red | Southern Biotech, Birmingham, AL |

**Table S2:** Antibodies used in the Flow cytometry analysis - Panel 2

| **Specific antibodies** | **Isotype/fluorochrome** | **Source** |
| --- | --- | --- |
| CD3 AF700 | Mouse IgG1 AF700 | Southern Biotech, Birmingham, AL |
| Bu 1-PE | Mouse IgG1 PE | Southern Biotech, Birmingham, AL |
| Monocyte/Macrophage FITC | Mouse IgG1 FITC | Southern Biotech, Birmingham, AL |
| TCRγδ- Biotin | Mouse IgG1 Biotin  Conjugate: SA PE Texas red (1:750) | Southern Biotech, Birmingham, AL |
